# An Integrated Mass Spectrometry and Molecular Dynamics Simulations Approach Reveals the Spatial Organization Impact of Metal-Binding Sites on the Stability of Metal-Depleted Metallothionein-2 Species

**DOI:** 10.1021/jacs.1c05495

**Published:** 2021-09-03

**Authors:** Manuel David Peris-Díaz, Roman Guran, Carmen Domene, Vivian de los Rios, Ondrej Zitka, Vojtech Adam, Artur Krężel

**Affiliations:** † Department of Chemical Biology, Faculty of Biotechnology, University of Wrocław, F. Joliot-Curie 14a, 50-383 Wrocław, Poland; ‡ Department of Chemistry and Biochemistry, Mendel University in Brno, Zemedelska 1, 613 00 Brno, Czech Republic; § Central European Institute of Technology, Brno University of Technology, Purkynova 123, 612 00 Brno, Czech Republic; ∥ Department of Chemistry, University of Bath, Claverton Down, Bath BA2 7AY, United Kingdom; ⊥ Department of Chemistry, University of Oxford, Oxford OX1 3TA, United Kingdom; # Functional Proteomics, Department of Cellular and Molecular Medicine and Proteomic Facility, Centro de Investigaciones Biológicas (CIB-CSIC), Ramiro de Maeztu 9, 28040 Madrid, Spain

## Abstract

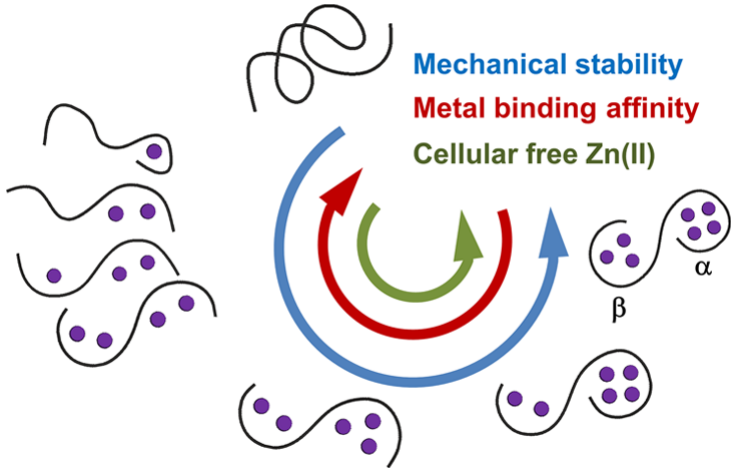

Mammalian metallothioneins (MTs) are a group of cysteine-rich proteins that bind metal ions in two α- and β-domains and represent a major cellular Zn(II)/Cu(I) buffering system in the cell. At cellular free Zn(II) concentrations (10^–11^–10^–9^ M), MTs do not exist in fully loaded forms with seven Zn(II)-bound ions (Zn_7_MTs). Instead, MTs exist as partially metal-depleted species (Zn_4–6_MT) because their Zn(II) binding affinities are on the nano- to picomolar range comparable to the concentrations of cellular Zn(II). The mode of action of MTs remains poorly understood, and thus, the aim of this study is to characterize the mechanism of Zn(II) (un)binding to MTs, the thermodynamic properties of the Zn_1–6_MT2 species, and their mechanostability properties. To this end, native mass spectrometry (MS) and label-free quantitative bottom-up and top-down MS in combination with steered molecular dynamics simulations, well-tempered metadynamics (WT-MetaD), and parallel-bias WT-MetaD (amounting to 3.5 μs) were integrated to unravel the chemical coordination of Zn(II) in all Zn_1–6_MT2 species and to explain the differences in binding affinities of Zn(II) ions to MTs. Differences are found to be the result of the degree of water participation in MT (un)folding and the hyper-reactive character of Cys21 and Cys29 residues. The thermodynamics properties of Zn(II) (un)binding to MT2 are found to differ from those of Cd(II), justifying their distinctive roles. The potential of this integrated strategy in the investigation of numerous unexplored metalloproteins is attested by the results highlighted in the present study.

## Introduction

Mammalian metallothioneins (MTs) are small (∼6–7 kDa) cysteine-rich proteins that participate in the metabolism of Zn(II) and Cu(I). Besides essential metal ions, MTs also bind toxic metal ions, such as Cd(II), Pb(II), and Hg(II), limiting their negative effects for cells.^1−5^ There are at least a dozen MTs isoforms (MT1–MT4 and their subisoforms) found in the cytosol, nucleus, mitochondria, and the extracellular environment,^1,6,7^ which differ in their metal-binding properties and their tissue localization.^1,5,8^ MT1 and MT2 are ubiquitously expressed, while MT3 and MT4 are present in the central nervous system and in stratified epithelial tissue, respectively.^1^ MTs are 60- to 68-amino acids long and form a dumbbell-shape polypeptide with two thiol-rich regions, separated by a conserved KKS linker containing 11 and 9 cysteinyl residues, designated α- and β-domains.^1−5^ The C-terminal α-domain (residues 31–61 in MT2) encloses a Zn_4_(Cys)_11_ cluster formed by five bridging and six terminal sulfur donors. In contrast, the N-terminal β-domain (residues 1–30), which includes a Zn_3_(Cys)_9_ cluster, is made up of three bridging and six terminal ligand donors.^9^ The 20 cysteinyl sulfur donors provide a tetrahedral coordination environment around each Zn(II). For decades, it was believed that all Zn(II) ions bound to MTs had identical low picomolar affinity.^10,11^ However, a decade ago, using highly sensitive zinc fluorescent probes, it was demonstrated that particular Zn(II) ions bind to MT2 with significantly different affinities varying from nanomolar to picomolar.^12^ Subsequently, similar differential binding properties of Zn(II) in human MT3 were postulated.^13,14^ This breakthrough revealed that under cellular conditions, where free Zn(II) concentration varies from 10^–11^ to 10^–9^ M, MTs exist as partially Zn(II)-depleted species and their speciation depends, inter alia, on the fluctuations of free Zn(II) and apoprotein expression or induction.^15−18^ Depending on the fluctuations of Zn(II) concentrations, MTs act as a molecular sponge; upon Zn(II) changes, MTs operate as Zn(II) buffers by either capturing excess Zn(II) or by donating Zn(II) to apoproteins which eventually restore the cellular free Zn(II) concentration.^12,15^ Although a considerable amount of physicochemical data have been generated since the discovery of MTs over 60 years ago, progress on structural characterization based on traditional biophysical methods has been sluggish.^3,19,20^ Structurally, MTs lack aromatic amino acids and secondary structures, and they exist in disordered forms. Therefore, most of the traditional biophysical methods including UV–vis, CD, NMR, or X-ray crystallography have failed to detect subtle differences among Zn(II)-depleted species or explain their mechanisms of formation. Moreover, the electronic configuration of Zn(II) (d^10^), high-energy charge-transfer transitions, and lack of NMR active nuclei hamper Zn(II) binding sites characterization using spectroscopic techniques. Thus, the majority of the biophysical data available for MTs have been recorded on Cd(II)-substituted species since this metal ion renders much better spectroscopic properties including the presence of NMR active ^111^Cd and ^113^Cd nuclei.^21^ However, at present, it is more evident that Cd(II) cannot be used indiscriminately as a Zn(II) probe in MT studies because it binds with high cooperativity to MT domains, in contrast to Zn(II).^22−26^ Even though Cd(II) ions bind to MTs with higher affinity than Zn(II), their stability constant values group differently, indicating distinct mechanisms of protein folding for each metal ion.^1,2,11,24^ In other studies where Co(II) was used as a spectroscopic probe to investigate the metal-binding mechanism in MTs, four Co(II) ions were found bound to the apoprotein, both in the α- and β-domains, and it was concluded that these ions bind independently prior to the formation of any cluster.^27−31^ Altogether, it has been demonstrated that despite the presence of the same number of metal ions in fully loaded MT states, different mechanisms drive the formation of metal-MT species. These mechanisms are currently not fully understood and are one focus of this study.

During the last two decades, mass spectrometry (MS), and in particular electrospray ionization mass spectrometry (ESI-MS), has become an indispensable technique in the studies of various MTs, as reflected in the number of publications.^8,32−37^ ESI-MS preserves the noncovalent metal–protein interactions, and it is likely that the solution-phase populations and conformational states are retained.^38−41^ A suitable solution concentration that minimizes ESI-induced pH changes, soft ESI conditions, and transmission parameters allows kinetic trapping of native-like protein states.^42−46^ Cysteinyl chemical labeling combined with MS has been used to study the binding of Cd(II), Zn(II), and other metals ions to MTs,^32,33,47−53^ and conformational studies have been performed by ion mobility mass spectrometry (IM-MS) experiments.^35,36,47,50^ However, despite the many advances made, the structural features of the Zn(II) binding sites in partially Zn(II)-depleted/loaded MT species are still under debate. To date, four reports have addressed mechanisms of Zn(II) binding to MT2.^32,33,35,37^ In the first study, it was concluded from a combined bottom-up MS and Cys labeling with iodoacetamide (IAM) approach that Zn(II) binds sequentially to the α-domain of MT2 until an α-Zn_4_MT2 cluster is formed. In a bottom-up MS experiment, proteins are digested in solution with an enzyme, and the resulting peptides are analyzed by MS. A degree of protection of the β-domain from alkylation was also observed.^32^ The same study concluded that a weakly bound Zn(II) ion is transferred from the β-domain in Zn_7_MT2 to the apo-form of sorbitol dehydrogenase (SDH). In the second study using ESI-MS, it was postulated that four Zn(II) ions bind independently to the protein.^37^ In this model, Zn(II) ions do not form any Zn_*x*_(Cys)_*y*_ cluster until the protein is saturated with seven Zn(II) ions. These conclusions were obtained from interpretation of limited experimental data from some ESI-MS signals and the derived noncooperativity patterns. In the third study, it was concluded from top-down MS and IM-MS experiments that the sequence Asn18-Cys38 of the apoprotein coordinates the first four Zn(II) ions and that the N- or C-termini interact weakly with Zn(II).^35^ In a top-down MS approach, intact protein is directly introduced into the gas phase, fragmented, and analyzed in the mass spectrometer. Although an attempt was made to localize Zn(II) binding sites, the top-down MS approach did not provide enough coverage. In the fourth study, we made use of a dual labeling strategy combined with a bottom-up MALDI-MS approach, and a partial redistribution of the four Zn(II) ions between the α- and β-domains was suggested.^33^ In agreement with another report,^35^ the region Cys21-Lys30 was identified to bind Zn(II) in the Zn_4_MT2 species. While altogether these studies evidence the noncooperativity nature of Zn(II) binding to either domain, the exact location and coordination features of the metal remain unclear. In this scenario, further high-resolution studies to characterize the Zn(II)-loading states in MT proteins and the associated binding mechanisms are necessary.

Integration of MS with molecular dynamics (MD) simulations has been reported in the study of other biological systems.^54−56^ Here, native MS, proteomics approaches including label-free quantitative bottom-up and top-down MS, and computer simulations including steered MD and several variants of metadynamics have been combined to determine the spatial organization of the MT proteoforms with the highest resolution reported until now. Our study provides crucial evidence about the mechanisms driving the formation of partially loaded MT species and explains the bases for metal affinity differentiation which results in their critical impact on Zn(II) buffering properties of MTs.

## Experimental Section

### Materials

All the reagents used were purchased from Merck, Acros Organics, Roth, BioShop, VWR International (Avantor), and Iris-Biotech GmbH. Milli-Q water (Merck) was used to prepare the buffers and solutions. The reagents were incubated with Chelex 100 resin (Bio-Rad) to eliminate metal ion trace contamination. More information about the reagents can be found in the Supporting Information.

### Expression and Purification of Metallothionein

MT2 (Addgene plasmid ID 105693) was overexpressed and purified in a bacterial system as described in the Supporting Information.

### Reactions of Zn_7_MT2, Cd_7_MT2, Zn_4_MT2, and Cd_4_MT2 with the IAM Alkylation Reagent

Cysteine residue profiling was performed by incubating 15 μM protein (Zn_7_MT2, Zn_4_MT2, Cd_7_MT2, or Cd_4_MT2) in 50 mM ammonium acetate at pH 7.4 with 1–10 mM IAM in the presence of 1 mM TCEP for 15 min at 25 °C in darkness. A 12 μL aliquot was pipetted and purified through ZipTip μ-C18, and the protein was eluted by adding 5 μL of Milli-Q water/acetonitrile (MeCN) solution (50:50, v/v).

### Dual Labeling Methodology for the Mapping of Zn(II)-Binding Sites

To directly map cysteine-rich binding sites, our previous MS-based dual labeling strategy was employed.^33^ First, the apoMT2 form was obtained by purifying Zn_7_MT2 using SEC with 10 mM HCl. The concentration of apoMT2 was then estimated by DTNB and Cd(II) titration experiments. To obtain the partially metalated Zn_1–7_MT2 proteins, the apoMT2 form was saturated with 1–7 ZnSO_4_ equiv under a nitrogen blanket in the presence of 1 mM TCEP at pH 7.4. This step was followed by buffer exchange to 50 mM ammonium acetate at pH 7.4. Then, to purify the samples, they were subjected three times to 10 min of spin time, using a 3 kDa Amicon ultra-4 centrifugal filter, purging nitrogen and adding 1 mM TCEP at pH 7.4 in each round. Partially metalated proteins were subsequently analyzed by ESI-MS. Then, a 15 μM Zn_0–7_MT2 aliquot was incubated in 1 mM IAM for 15 min at 25 °C in darkness. From this, an aliquot was purified by ZipTip μ-C18, eluting with 5 μL of Milli-Q water/MeCN solution (50:50, v/v), and measured by MALDI-MS, and another aliquot was analyzed by ESI-MS. The remainder of the sample (80 μL) was subjected to the dual labeling strategy. First, the pH was reduced with 0.1% FA and 1 mM DTT, and the IAM excess was removed by purification with a C18 resin. The eluted protein was doubly labeled by incubation in 3 mM *N*-ethylmaleimide (NEM) for 30 min, at 25 °C. Subsequently, an aliquot was analyzed by MALDI-MS. The rest of the sample underwent a bottom-up LC-MS approach in a solution digestion using trypsin at a weight ratio of 1:20 for 30 min at 37 °C, and the reaction was quenched by the addition of 5 μL of 0.1% formic acid.

### Native MS and Native Top-Down MS

A quadrupole time-of-flight (qTOF) Bruker Maxis Impact mass spectrometer (Bruker Daltonik GmbH, Bremen, Germany) calibrated with ESI-TOF Tuning Mix (Sigma-Aldrich) was used for the ESI-MS measurements. The samples were infused with a 3 μL/min flow rate, and the spectra were recorded in positive mode. The parameters were set up to preserve the Zn(II)-protein complexes: capillary voltage of 3.5 kV, CID energy of 10 eV, isCID 20 eV, collision cell transfer time of 120 μs, prepulse storage of 25 μs, end plate offset potential of 500 V, nebulizer gas (N_2_) pressure of 1.5 bar, drying gas (N_2_) flow rate of 5 L/min, and drying temperature of 180 °C. To prevent oxidation of free thiols, a low capillary voltage was used in addition to 1 mM TCEP (pH 7.4) and a nitrogen blanket during the sample preparation. TCEP was used not only as a reducing agent but also because it binds Zn(II) less tightly than DTT (submillimolar affinity). At neutral pH, the thiol group tends to oxidize. However, the presence of 1 mM TCEP prevented its oxidation. Under these conditions, Cys oxidation was not observed (commonly identified by the loss of 2 Da per Cys residue to form a cysteine thioaldehyde). The mass range was set from 500 to 3000 *m*/*z* at a 1 Hz acquisition rate, recorded, and averaged over 1 min. To perform a native top-down analysis, the precursor ion was subjected to quadrupole mass filtering and a cycle of increasing collision energies (10–60 eV). The data were analyzed with the Bruker Compass data analysis software package and with the MetaOdysseus R-package.^57^

### MALDI-MS

MALDI-MS experiments were performed on a MALDI-TOF/TOF MS Bruker UltrafleXtreme instrument (Bruker Daltonik GmbH, Bremen, Germany). The MALDI-TOF matrix used was 2,5-dihydroxybenzoic acid (DHB) prepared in 30% MeCN and 0.1% trifluoroacetic acid. The measurements were performed in a linear positive mode, with a 2–20 kDa range. The spectra were acquired by averaging 2000 spectra obtained from 2000 laser shots per spot. The laser power was set to 5–10% above the threshold. The instrument was calibrated using a protein calibration mixture from Bruker Daltonik GmbH, Bremen, Germany and controlled by the software flexControl v 3.4 (Bruker Daltonik GmbH, Bremen, Germany).

### Electronic Absorption Spectroscopy

UV spectra were recorded on a JASCO V-670 spectrophotometer at 25 °C with a 1 cm quartz cuvette in the range of 200–300 nm. Titrations of 1 μM apoMT2 were performed in chelexed 50 mM borate buffer (100 mM NaClO_4_, pH 7.4) with 500 μM ZnSO_4_ or CdSO_4_ under anaerobic conditions. Spectra were averaged from the following three accumulations.

### Label-Free Bottom-up MS-Based Proteomics

NanoLC-MS/MS experiments were performed using an Easy-nLC 1000 nanosystem coupled to a Q Exactive hybrid quadrupole-Orbitrap mass spectrometer (Thermo Fisher Scientific). Four μL of each sample were injected, loaded into an Acclaim PepMap 100 precolumn (Thermo Fisher Scientific), and eluted in a RSLC PepMap C18 with a 50 cm length, 75 μm inner diameter, and 2 μm particle size (Thermo Fisher Scientific). The mobile phase flow rate was 300 nL/min using 0.1% formic acid in water (solvent A) and 0.1% formic acid and 100% acetonitrile (solvent B). The gradient profile was set as follows: 5–35% solvent B for 90 min, 35%–100% solvent B for 4 min, and 100% solvent B for 8 min. The spray voltage was 1.9 kV, and the capillary temperature was 270 °C. A full MS scan was acquired (target automatic gain control (AGC) using a value of 3 × 10^6^, maximum injection time of 100 ms and resolution of 70,000 at *m*/*z* 200), and tandem MS was performed with a data-dependent method using the top 15 most intense precursor ions. Fragmentation of the ions was performed with a normalized collision energy of 27 eV. MS/MS scans were acquired with a starting mass of *m*/*z* 50, target AGC of 2 × 10^5^, resolution of 17,500 (at *m*/*z* 200), intensity threshold of 8 × 10^4^, isolation window of 2.0 *m*/*z* units and maximum IT of 100 ms. A dynamic exclusion time of 20 s was used to discriminate against previously selected ions.

### MS Identification and Label-Free Quantification

MS data were analyzed with Proteome Discoverer (version 2.4.1.15) (Thermo Fisher Scientific) using standardized workflows. Mass spectrum *.raw files were searched against a custom protein database listing, among others things, the MT2 fasta file using MS Amanda with trypsin(semi) selected as an enzyme.^58^ Precursor and fragment mass tolerances were set to 5 ppm and 0.02 Da, respectively. This allowed three missed cleavages, methionine oxidation, carbamidomethyl, and NEM of cysteines as variable modifications. A fixed-value PSM validator of 0.05 was employed. To perform the relative label-free quantification, the Feature Mapper and Minora Feature detector modules were used. Chromatographic alignment was performed using a maximum RT shift of 10 min and a 5 S/N minimum threshold. The NEM or IAM content in particular Cys residues was calculated as follows. First, the peptide sequences were aligned to the protein sequence. Then, the total abundance of Cys-NEM and Cys-IAM labeling was calculated for each individual Cys residue. Finally, the abundances for each Cys residue were normalized considering that the total abundance is the sum of Cys-NEM and Cys-IAM.

### Computational Studies

A summary of the systems and the methods employed is presented in Table 1.

**Table 1:** Summary of the Simulation Systems and Methods Employed in This Study

set	system	method	no. of runs	collective variable	simulation time (ns)
1	Zn_7_MT2	SMD with fixed C-terminal	100^a^	end-to-end distance	170
2	Cd_7_MT2		100^a^		170
3	β-Zn_3_MT2		100^a^		170
4	Zn_7_MT2	SMD with fixed N-terminal	100^a^		170
5	Zn_7_MT2	classical MD	2	–	1000
6	β-Zn_3_MT2	classical MD	2	–	1000
7	Zn_7_MT2	WT-PBMetaD	1	seven CN Zn–S	700
8	Zn_7_MT2	WT-MetaD	28^b^	Zn–S distance	100
9	β-Zn_3_MT2		12^b^		50
				total simulation time (ns)	3530

a Stands for the number of independent replicates of the same simulation system.

b Refers to simulation runs where the collective variable considered different Zn(II) ions and/or S atoms.

#### Model Building

The initial structure for the computational studies was selected to be the X-ray structure PDB ID 4MT2 that contains four Cd(II) and two Zn(II) ions. In the cell, MTs exist as homogeneous Zn(II) proteins, but for the above-mentioned reasons, their Cd(II) counterparts and isolated β-domain were used in the biophysical studies. Initial Zn_7_MT2, β-Zn_3_MT2 and Cd_7_MT2 systems were set up by replacing all the metal ions in the crystal structure with Zn(II) or Cd(II), respectively. In these models, each Zn(II) or Cd(II) ion occupied a binding site coordinated by four deprotonated Cys residues. The protonation and tautomeric state of residues at pH 7.0 were assigned using PROPKA. Cys residues were modeled in the deprotonated form, as this is how they are found experimentally, and in agreement with the PROPKA results. The AMBER FF19SB force field was used to model the protein including AMBER parametrization for Zn(II) and the coordinating residues^59^ and a parameter compatible with the AMBER force field for Cd(II).^60^

#### Steered Molecular Dynamics Simulations

Constant-speed steered molecular dynamics (SMD) simulations were used to study the unfolding dynamics of Zn_7_MT2, β-Zn_3_MT2, and Cd_7_MT2. Each protein was solvated in a rectangular box with TIP3P water molecules, and NaCl was added to achieve electrical neutrality. The simulation box was set at 36 × 32 × 720 Å^3^, to warrant that the distance in the pulling axis is longer than twice the maximum pulling distance to avoid any issues with the periodic images.

Each system was subjected to 10,000 steps of steepest-descent minimization, followed by a temperature ramp from 0 to 300 K in the NVT ensemble. The temperature was controlled using a Langevin thermostat with a damping coefficient of 1 ps^–1^. The particle mesh Ewald method was used to calculate the long-range electrostatic interactions using a cutoff of 10 Å. The SHAKE algorithm was used to constrain bonds involving hydrogen atoms to allow a time step of 2 fs. Subsequently, 200 ns in the NPT ensemble at 300 K and 1 atm followed. Isotropic position scaling was used with a relaxation time of 2 ps. The pressure was kept constant using a Berendsen barostat during the equilibration and the Monte Carlo barostat during production runs as implemented in the AMBER software. SMD simulations were performed using the AMBER16 software. Twenty snapshots at equal intervals were extracted from the last 2 ns of the 200 ns classical MD trajectories of each system. Then, five independent SMDs runs at constant speed were performed for every snapshot starting with randomized initial velocities, amounting to 100 runs. The pulling force was applied to the CA atom of the Met1 residue at the N-terminus. In order to remove irrelevant degrees of freedom such as translations and rotations and to move only along the intended axis, a 2 kcal·mol^–1^ positional restraint was applied to the CA atom of the last residue, Ala61. The metal dissociation mechanism was found to be independent of the tethering geometry while performing SMD simulations with the N-terminal fixed. The force constant selected was strong enough to observe a linear relationship between the end-to-end Met1(CA)-Ala61(CA) distance and the selection of the positional constraint. At the same time, the magnitude was such that high fluctuations in the force profile were not observed. In particular, four different force constants of 1, 2, 3, and 5 kcal·mol^–1^ (pN·Å^–1^) were tested. The 3 kcal·mol^–1^ choice was found to be strong enough for the required purposes, and it did not produce high fluctuations in the rupture force profile. The effect of the pulling speed on the rupture force was considered by testing several pulling speeds: 10, 20, 50, 100, 300, 500 Å·ns^–1^. A pulling speed of 100 Å·ns^–1^ was chosen, as it rendered the fastest regime from the range of pulling speeds with comparable rupture forces to the lowest speed considered (10 Å·ns^–1^). Initial trials established the maximum pulling length required to induce the transition from a fully folded to a fully extended structure. For instance, for Cd_7_MT2 and Zn_7_MT2, the initial end-to-end Met1(CA)-Ala61(CA) distance was 36 Å, and the maximum pulling length was chosen to be 205 Å. The simulation time needed to achieve such stretching at the pulling speed selected (100 Å·ns^–1^) was calculated as (*r*_F_ −*r*_0_)/*v*. In total 170 ns of SMD simulation time was considered for analysis.

To identify metal dissociation pathways, a contact number (CN) index was used to record the number of ligands contributing from the potential 20 Cys residues involved or water molecules coordinating a selected metal center, CN_Zn–S_ or CN_Zn–O_, respectively. The minimum value of the CN_Zn–S_ parameter is 0, and the maximum is 4 according to the values observed in proteins where Zn(II) or Cd(II) ions are coordinated by Cys residues.^61^ The software PLUMED 2.6^62^ was used to calculate the contact index number CN, which is defined as
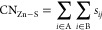
1where A is the metal ion, B corresponds to the Cys residues, and *s*_*ij*_ is a switching function. The switching function is defined as
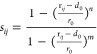
2where *n* = 8 and *m* = 12, and they define the steepness of the switching function. *r*_0_ and *d*_0_ were determined from the radial distribution function of Zn(II) in the protein and were set to 0.3 and 2.4 Å, respectively.

Then, a matrix that contained all the possible CN_Zn–S_ values for each of the seven Zn(II) ions considered was built for each frame of the trajectory and subsequently analyzed using an in-house R script. A Zn(II) ion was considered “free” when CN < 0.9. 100 independent SMD runs of 1.7 ns were analyzed. Once dissociation pathways had been characterized, the identical pathways were identified, and the probability of each identified pathway was calculated.

#### Metadynamics and Its Variants

The time scales of Zn(II) unbinding/binding to MT2 are not accessible with standard MD simulation, so enhanced sampling methods such as metadynamics are needed to sample this type of “rare” event.^63^ For the metadynamics calculations, only two of the previous systems were considered: Zn_7_MT2 and β-Zn_3_MT2. Their initial setup was almost identical to the one described earlier with the exception of the simulation box that was set at 62 and 48 Å^3^, respectively, for Zn_7_MT2 and β-Zn_3_MT2, and the LINCS algorithm was employed in order to be able to use a 2 fs time step. A position restraint was applied to the heavy atoms of the protein for the NVT equilibration. Subsequently, 10 ns at constant pressure and temperature (NPT) was used. Berendsen weak coupling was used to keep the pressure isotropically at 1 bar during the equilibration, and the temperature was set at 300 K. Then, 500 ns of production run was collected in the absence of any restraints using the Parrinello–Rahman barostat to isotropically control the pressure at 1 bar and the Nosé–Hoover thermostat to maintain the temperature at 300 K. Two independent replicas were run for each system, amounting to 2 μs of dynamics. In the time scales of these simulations, dissociation events were not recorded. The metadynamics computations were performed with GROMACS 2018.4^64^ in combination with the PLUMED plugin.^62^ The input files were deposited in PLUMED-NEST repository (https://www.plumed-nest.org/eggs/21/032/).

#### Well-Tempered Parallel-Bias Metadynamics

WT-PBMetaD was used to study the spatial organization of metal ions in partially Zn(II)-loaded states in Zn_7_MT2. PBMetaD is an extension of metadynamics that allows biasing multiple collective variables (CVs) in parallel, and consequently it accelerates the convergence of the free-energy landscape.^65,66^ Seven CVs were employed, each of them describing the number of contacts between a Zn(II) ion and the sulfur atoms from the group of Cys residues to which they were initially bound (eqs 1 and 2). To speed up the calculation of the CNs, a neighbor list was used with an 8 Å cutoff updated every 100 steps. The cutoff and frequency for the recalculation of the list were carefully chosen by comparing the results to those in the absence of any cutoff. The chosen CVs successfully described the dissociation/association process, and the seven-dimensional space was fully explored. The metadynamics bias potential was built using an initial height of 0.6 kcal·mol^–1^, and a width of 0.12. Gaussians was deposited every 500 time steps. The bias factor was set to 18. To enhance the convergence, four multiple walkers were used. For each system, ∼0.7 μs of dynamics was recorded, which corresponds to a time when all of the states have been visited multiple times. The convergence of the free energy surface (FES) was assessed in two ways: (i) qualitatively by projecting the FES on each CV at different simulation times until convergence was reached, and (ii) quantitatively by performing an estimation of the error in the free energies using block analysis of the weighted histogram from the biased CVs. The unbiased or weighted histograms were obtained by an umbrella sampling-like reweighting algorithm.^67^ The error of the free energy was estimated using a block analysis protocol. The dependency of the average free energy error on the block size was considered; it was found that as the block size increased, the error associated with the free energy increased until a plateau was reached. The magnitude of the average free energy error was found similar regardless of the protocol employed for the analysis, either using a unique concatenated trajectory or individual trajectories from each walker. Reweighted free-energy maps were obtained as a function of 2 CVs that were not directly biased. To obtain CN_Zn(II)-MT2_, which described the total Zn(II) ion bound to MT2, the CN_Zn–S_ for each Zn(II) ion in the Zn_7_MT2 system was first calculated. Then, the values were filtered using eq 2 with *r*_0_ and *d*_0_ set to 2.5 and 0 Å, and finally the seven CN were combined. The CV CN_Zn–O_ gives the CN between each Zn(II) ion from the Zn_7_MT2 system and the oxygen atoms from the water molecules using eq 1 and a similar switching function *s*_ij_ to that in eq 2. The parameters of the switching function were set to *n* = 12, *m* = 24, *r*_0_ = 2.9 Å and *d*_max_ = 5 Å. The CV dRMSD was employed to calculate the distance root-mean-square deviation (dRMSD) with respect to the folded structure. The RMSD was calculated for pairs of atoms that were within 0.1–8 Å.

#### Well-Tempered Metadynamics

WT-MetaD^68^ was used to estimate the Zn–S bond dissociation energy of each of the metal sites in the Zn_7_MT2 and β-Zn_3_MT2 systems. In order to do this, a CV that described the distance between the Zn(II) ion and the S atom of each Cys residue was selected. A bias factor of 18, initial Gaussian height of 0.6 kcal·mol^–1^ and width of 0.12 Å were selected. Gaussians were deposition every 500 time steps. Eight multiple walkers were employed to facilitate convergence. To limit the region to be explored and speed up convergence, an upper wall was set up at 4 Å, which corresponds to a distance where any Zn–S interaction is negligible. Potential fictitious effects of the wall were investigated by extending the wall up to 8 Å in a trial run. Convergence was monitored as described earlier for the WT-PBMetaD calculations.

## Results and Discussion

**Scheme 1:**
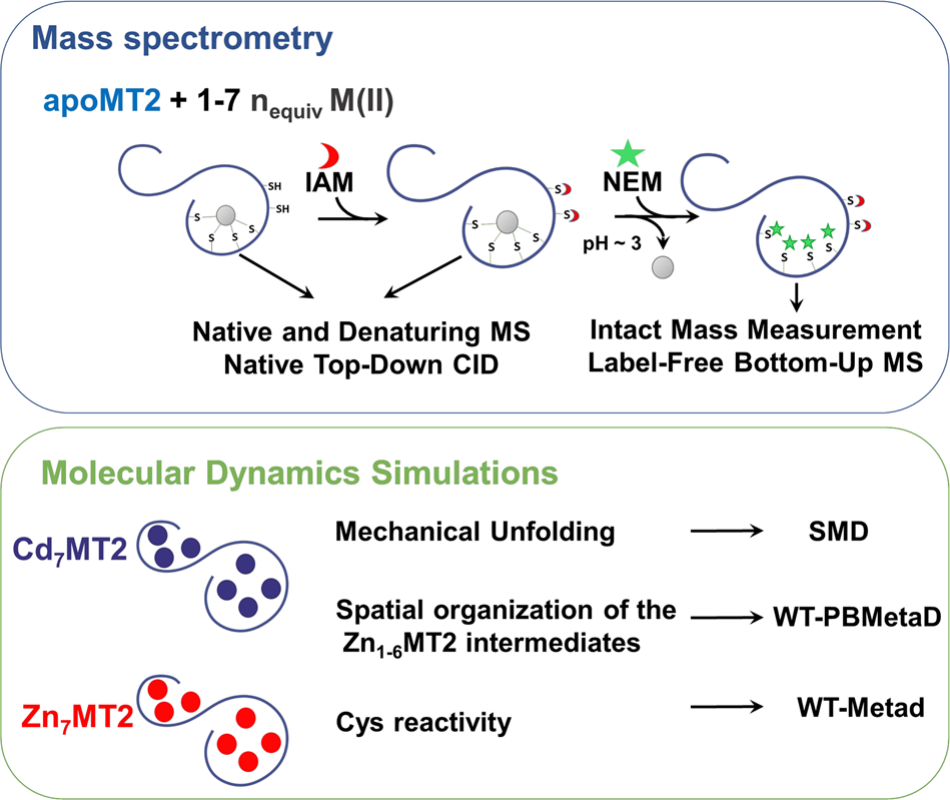
Overview of the Mass Spectrometry and Computational Approaches Employed in This Study Native top-down CID refers to the measurement of the mass of cleavage protein backbone after a protein under native solution conditions is subjected to collision-induced dissociation. In the bottom-up MS experiment, the protein is digested using trypsin, and the resulting peptides are analyzed with nanoLC-MS/MS. The Cys-IAM and Cys-NEM labeled residues were identified by ∼57 Da and ∼125 Da mass shifts, respectively. Abbreviations: CID, collision-induced dissociation; IAM, iodoacetamide; NEM, *N*-ethylmaleimide; *n*_equiv_, the number of Zn(II) or Cd(II) molar equivalents; SMD, steered molecular dynamics simulations; WT-PBMetaD, well-tempered parallel-bias metadynamics simulations; and WT-Metad, well-tempered metadynamics simulations.

In order to label free Cys residues, experiments were performed to optimize the IAM concentrations aimed at maintaining the interactions between Zn(II) ions and holoMT2 (Zn_7_MT2) as well as the 20 Cys residues labeled in the apoMT2 form. Once a quantity was optimized, this amount of IAM was added to the apoMT2 system, which was incubated with 0–7 Zn(II) equiv. Mass spectra were recorded under native MS and denaturing conditions by ESI-MS and MALDI-MS. Incubation of the apoMT2 system with increasing Zn(II) equiv yielded a linear decrease of the Cys-IAM labeled until 6 Zn(II) equiv was reached, confirming that Cys residues were labeled by IAM independently of the protein conformational change upon Zn(II) binding. Subsequently, the moieties labeled by IAM and NEM were identified using a bottom-up LC-MS approach. In addition, a label-free quantitative bottom-up proteomics analysis was performed to determine the contribution of each of the 20 Cys residues to the Zn(II) binding mechanism. The metal binding mechanism was followed by monitoring changes in the molar absorption coefficients obtained from spectrophotometric titrations of apoMT2 with Zn(II) and Cd(II). Complementing this analysis, to probe whether Zn(II) ions are redistributed in the α- and/or β-domains, chemical labeling and collision-induced dissociation experiments on Zn_4_MT2 and Cd_4_MT2 were performed (Scheme 1).

### A Label-Free Bottom-Up MS to Quantitatively Characterize the Zn(II)-MT2 Binding Mechanism

**Figure 1:**
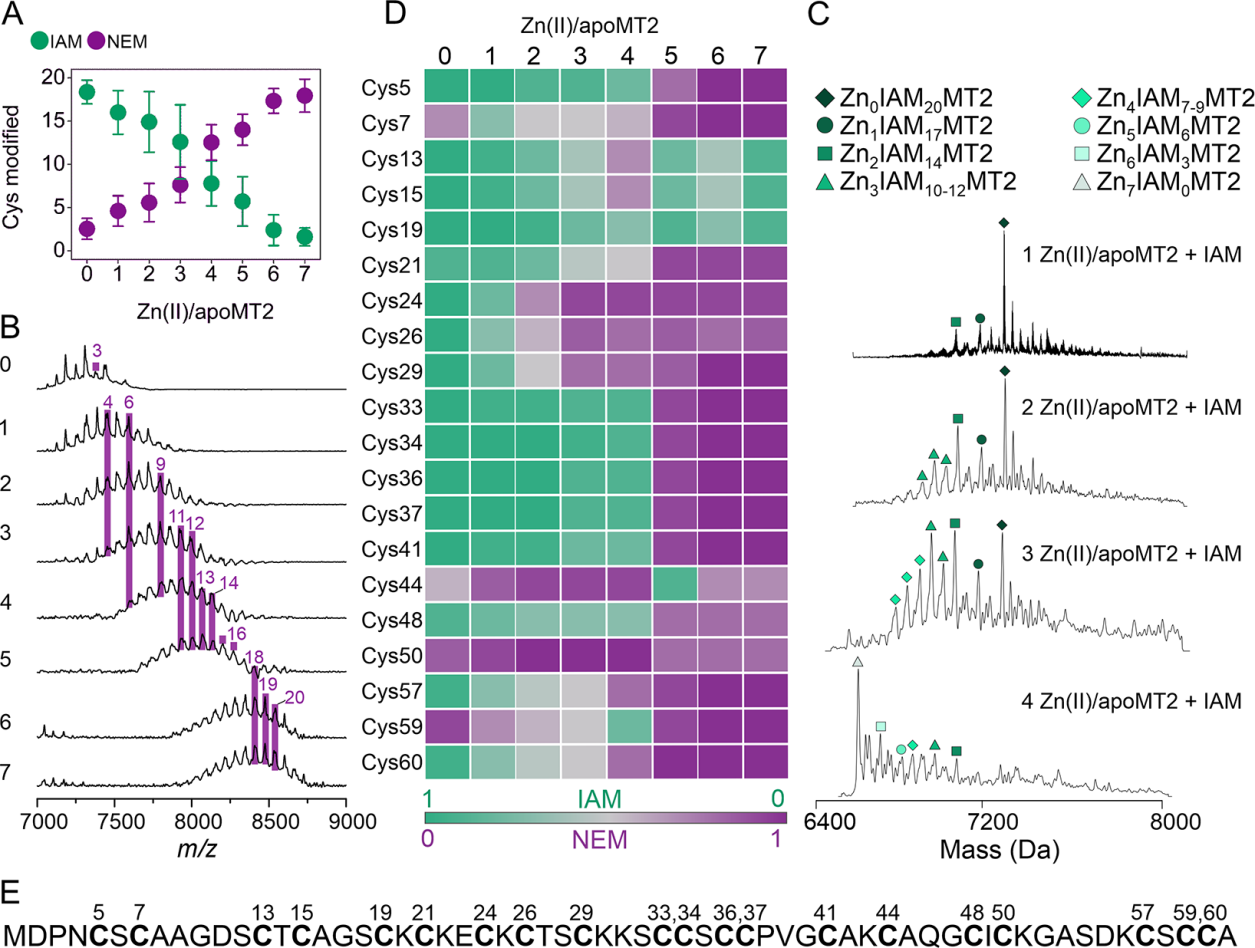
(A) Cysteine profiling for apoMT2 with 0–7 equiv of Zn(II) by a dual labeling strategy utilizing IAM and NEM and (B) monitored with MALDI-MS. The Cys-IAM and Cys-NEM labeled residues were identified by a ∼57 Da and ∼125 Da mass shift, respectively. (C) Native MS spectra for the Zn(II):apoMT2 complex formed after the addition of 1–4 Zn(II) equiv followed by incubation with 1 mM IAM in darkness (25 °C, 15 min). (D) Label-free bottom-up proteomic analysis of apoMT2 with 0–7 Zn(II) equiv that had been dual-labeled with IAM and NEM. First, the Zn(II):apoMT2 complex was formed, followed by incubation with IAM (1 mM, 15 min, 25 °C). The excess IAM and Zn(II) were removed with C18 purification, and the protein was labeled with NEM (3 mM, 30 min, 25 °C). Finally, the double-labeled proteins were trypsinized and analyzed using a nanoLC-MS/MS system. (E) Human MT2 sequence indicated the Cys residues.

The IAM-labeled apoMT2 protein showed 17–20 modifications (Figure 1A,B). This range of modifications was also observed in the native mass spectra (Figure 1C, Table 2, and Table S1). Double-labeled peaks were observed in the evolution of IAM_20_NEM_0_MT2 to IAM_17_NEM_3_MT2 (Figure 1A,B). Cys59, Cys50, and Cys44 were found to be bound to NEM and not IAM (Figure 1D,E) after analysis of the NEM/IAM relative intensities.

**Table 2:** Chemical Labeling of Free and Zn(II)-Bound Cys Residues for Different Zn(II)-Depleted MT2 Species Followed by MALDI-MS and ESI-MS

Zn(II) equiv	species (ESI-MS)	free Cys-IAM residues (native ESI-MS)	free Cys-IAM residues modified (MALDI-MS)	Zn(II)-bound Cys-NEM residues modified (MALDI-MS)
0	Zn_0_MT2	Zn_0_IAM_18–20_MT2	IAM_17–20_MT2	IAM_17–20_NEM_3–0_MT2
1	Zn_0–2_MT2	Zn_0_IAM_19–20_MT2	IAM_11–20_MT2	IAM_11–20_NEM_9–0_MT2
		Zn_1_IAM_17_MT2		
		Zn_2_IAM_14_MT2		
2	Zn_0–3_MT2	Zn_0_IAM_19–20_MT2	IAM_11–20_MT2	IAM_11–20_NEM_9–0_MT2
		Zn_1_IAM_17_MT2		
		Zn_2_IAM_14_MT2		
3	Zn_0–4_MT2	Zn_0_IAM_19–20_MT2	IAM_8–20_MT2	IAM_8–20_NEM_12–0_MT2
		Zn_1_IAM_17_MT2		
		Zn_2_IAM_14_MT2		
		Zn_3_IAM_10–12_MT2		
		Zn_4_IAM_7–9_MT2		
4	Zn_3–5_MT2	Zn_3_IAM_10–12_MT2	IAM_6–13_MT2	IAM_6–13_NEM_14–7_MT2
		Zn_4_IAM_7–9_MT2		
		Zn_5_IAM_6_MT2		
		Zn_6_IAM_3_MT2		
		Zn_7_IAM_0_MT2		
5	Zn_5–7_MT2	Zn_5_IAM_6_MT2	IAM_9–2_MT2	IAM_9–2_NEM_11–18_MT2
		Zn_6_IAM_3_MT2		
		Zn_7_IAM_0_MT2		
6	Zn_5–7_MT2	Zn_5_IAM_6_MT2	IAM_2–0_MT2	IAM_2–0_NEM_18–20_MT2
		Zn_6_IAM_3_MT2		
		Zn_7_IAM_0_MT2		
7	Zn_6–7_MT2	Zn_6_IAM_3_MT2	IAM_2–0_MT2	IAM_2–0_NEM_18–20_MT2
		Zn_7_IAM_0_MT2		

The apoMT2 form was incubated with 1 Zn(II) equiv, and subsequently IAM was added to the Zn(II)-MT2 complex. The resulting IAM modification profile was centered at IAM_16_MT2, suggesting that 16 Cys residues are free and four Cys are bound to Zn(II) (Figure 1A,B, Figure S1A, and Table 2). This stoichiometry was confirmed by the presence of the Zn_1_IAM_16_MT2 complex in the native mass spectra (Figure 1C, Table 2, and Table S1). Furthermore, the dual-labeling experiment also showed four NEM-labeled Cys, confirming that a Zn(II) ion was bound to four Cys (Figure 1B) that were identified by analysis of the bottom-up MS data. Cys44 and Cys50 had an increase of Cys-NEM content at 1 Zn(II) equiv, suggesting that they bound Zn(II) (Figure 1D,E and Table 2). Analysis of the bottom-up data shows a slight increase in the NEM content of Cys residues from the β-domain region (Cys21-Cys29) (Figure 1D,E). There are two possible explanations for this observation: either there is a two-site competition or there are multiple Zn(II)-depleted states. The latter was supported by the presence of multiple Zn_0–2_MT2 species in the mass spectra (Figure 1C). An ion mobility-MS experiment reported a collision cross section (CCS) profile for Zn_1_MT2 with two conformers: a compact one (∼725–920 Å^2^) and an extended one (∼1000 Å^2^).^35^ The CCS values for unfolded apoMT2 and for Cd_4_MT2 provided a reference for two- and single-extended domains at ∼1050 and 900 Å^2^, respectively. Thus, considering these data, we may conclude that the first Zn(II) ion can be bound either to both domains corresponding to a compact conformer or to a single domain that corresponds to an extended conformer.

Upon addition of 2 Zn(II) equiv, the NEM/IAM abundance increased, which further supports our conjecture that a second Zn(II) ion can bind to the β-domain, in particular involving Cys24-Cys29 region (Table 2). These results suggest that both the α- and β-domains participate in metal-binding upon addition of the first 2 Zn(II) equiv. Zn_1_IAM_16_MT2 and Zn_2_IAM_14_MT2 species recorded in the ESI-MS were confirmed by MALDI-MS with the double modified proteins IAM_16_NEM_4_MT2 and IAM_14–13_NEM_6–7_MT2 (Figure 1B,C, Table 2, and Table S1). Accordingly, the Zn(II):Cys stoichiometry changed from 4:1 to 6:2 or 7:2 upon second Zn(II) binding. These numbers could also suggest a conformation where the two Zn(II) ions form a Zn_2_Cys_6_ cluster, and thus both conformations may coexist with similar folding free energies.

The first potential situation could be one where two Zn(II) ions are bound to residues from the same domain. The second potential situation could correspond to one where each Zn(II) ion is bound to a different domain. The Zn_1_IAM_16_MT2 and Zn_2_IAM_14_MT2 species and the double-modified proteins IAM_16_NEM_4_MT2 and IAM_14–13_NEM_6–7_MT2 are also observed (Figure 1B,C, Table 2, and Table S1), and thus the Zn(II):Cys stoichiometry changes from 4:1 to 6:2 or 7:2 upon a second binding event taking place. However, there is also the possibility of a configuration where the two Zn(II) ions form a Zn_2_Cys_6_ cluster. Spectrophotometric titrations of apoMT2 with Zn(II) and Cd(II) were performed to determine absorptive features as a function of the metal-binding process (Figure S2). Analysis of the increment difference absorption spectra obtained by subtracting successive spectra showed a decreased molar absorbance coefficient upon addition of 2 Zn(II) equiv (Table S2). This result suggests that the second Zn(II) does not form a new independent ZnCys_4_ site but supports the Zn_2_Cys_6_ cluster configuration. The opposite is Cd(II), which shows no change in the molar absorption coefficient for 1–2 Cd(II) equiv and indicates that it forms two CdCys_4_ sites.

Incubation of the apoMT2 form with 3 Zn(II) equiv led to an IAM modification profile accounting for 11–12 modifications. The IAM_11_NEM_9_MT2 peak suggests that three Zn(II) ions are coordinated by nine Cys residues (Figure 1A,B). The resulting native mass spectra also revealed Zn_3_IAM_11_MT2 among other species (Figure 1C, Table 2, and Table S1). Three new Cys residues coordinated Zn(II), and these were identified as Cys13, Cys15, and Cys21 (Figure 1D,E). Comparison of the MS/MS data reported for 2 and 3 Zn(II) equiv suggests that two Zn(II) ions are bound to the β-domain forming a Zn_2_Cys_6_ cluster and one Zn(II) ion is bound to the α-domain (Figure 1D,E and Table 2). The difference of molar absorption coefficient increases with respect to the value of the 2 Zn(II) equiv, suggesting that a new Zn(II) site has been formed (Figure S2 and Table S2).

After addition of 4 Zn(II) equiv to the apoMT2 system, the mass spectra revealed a Zn_4_IAM_9_MT2 product (Figure 1C, Table 2, and Table S1). Double-modified IAM_9_NEM_11_MT2 species were also observed, which confirmed that 11 Cys residues coordinate four Zn(II) ions (Figure 1A,B). Thus, two more Cys residues coordinate Zn(II) upon addition of the fourth Zn(II) equiv. The region Cys33-Cys41 from the α-domain was mostly modified by IAM, which suggested that four Zn(II) ions were not bound exclusively to the α-domain, and a Zn_2_Cys_6_ cluster is formed per domain (Table 2 and Figure 1D,E). A slight decrease in the molar absorbance coefficient from the 3 Zn(II) equiv was observed and interpreted as the formation of Zn(II) cluster (Figure S2 and Table S2).

After addition of 5 Zn(II) equiv to apoMT2, the protein binds Zn(II) ions through the Cys21-Cys41 region and, partially, the N- and C-terminal (Figure 1D,E). Two unoccupied Zn(II) binding sites, one at each domain, are inferred from the MS data recorded (Figure 1D,E and Table 2). In agreement, up to six free Cys residues (see Table 2) together with Zn_5_IAM_3_MT2 and Zn_5_IAM_6_MT2 are identified in the mass spectra; hence, between 14 and 17 Cys residues could coordinate five different Zn(II) ions (Figure 1A,B). The contribution from the sixth Zn(II) and seventh Zn(II) ions did not alter significantly the modification profile reported in Figure 1D,E. Overall, the Cys13-Cys19 residues from the β-domain are modified by IAM to a greater extent than the Cys44-Cys50 residues from the α-domain, suggesting that the last Zn(II) ion binds to the β-domain (Figure 1D,E and Table 2). The last 3 Zn(II) equiv results in lower difference molar absorption coefficients and evidence binding to MT2 forming bridging and terminal Zn–S bonds.

### Zn_4_MT2 Folds into a Two-Domain Structure Rather than into One Cluster-like Cd_4_MT2

**Figure 2:**
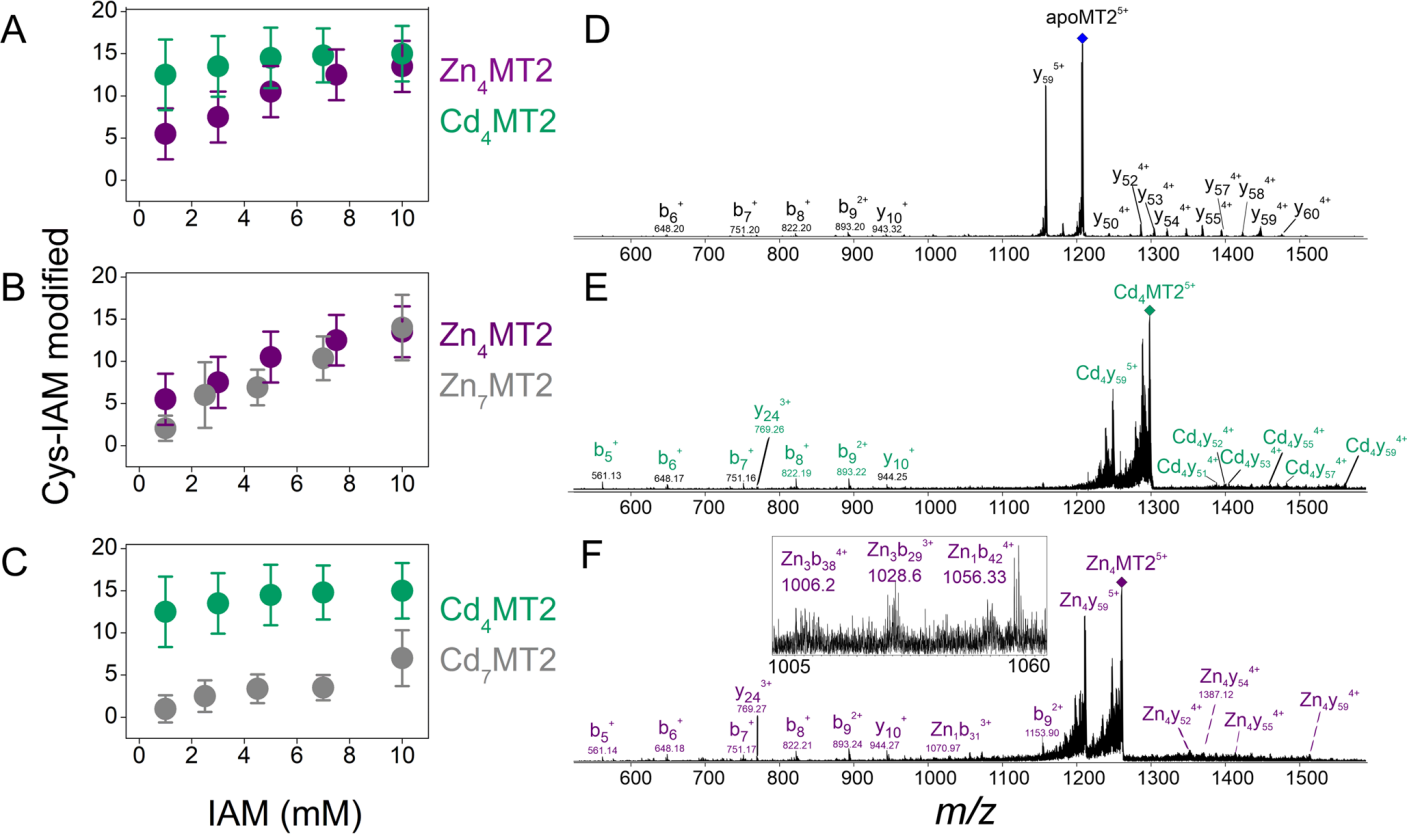
Chemical labeling and top-down MS of Zn_4_MT2 and Cd_4_MT2 to determine the location of Cd(II) and Zn(II) ions. (A–C) Cys residue modification dependence on the molar ratio (0–10 mM) of the alkylating reagent IAM monitored by MALDI MS. The protein complexes were formed by incubating apoMT2 with the appropriate metal ion concentration determined with DTNB and PAR assays. Then, the complex was analyzed with native MS to verify the metal-protein stoichiometry. CID spectra for (D) apoMT2, (E) Cd_4_MT2, and (F) Zn_4_MT2.

Cys labeling experiments on Zn_4_MT2 and Cd_4_MT2 proteins were performed to strengthen our results. The metal-protein complexes were prepared by addition of 4 Zn(II) or 4 Cd(II) equiv to apoMT2. Then the number of Cys residues modified at increasing IAM concentrations was monitored. Depending on the IAM concentration, IAM may not only label free Cys but it could also displace metal ions bound to Cys residues, and subsequently it could alkylate them. Upon addition of 1 mM IAM to the metal-protein complex, Cd_4_MT2 was found to have 12 Cys modified, in contrast to Zn_4_MT2, where only 5–6 Cys residues were modified (Figure 2A and Figure S3).

Upon increasing the IAM concentration, the number of IAM-labeled Cys residues increased in the Zn_4_MT2 system but remained constant in the Cd_4_MT2 system. This observation suggests that the distribution of the four Cd(II) and Zn(II) ions within the protein differs. It is well-known that Cd(II) binds with stronger affinity than Zn(II) to MT2, and it is proposed that four Cd(II) are located in the α-domain in the Cd_4_MT2 system. These observations are consistent with our data; addition of 1 mM IAM to Cd_4_MT2 modified the β-domain free Cys residues, and those in the α-domain remained mostly unmodified. Comparable experiments performed with Zn_7_MT2 showed a similar IAM concentration dependence as for Zn_4_MT2 (Figure 2B). A similar Cys modification profile for Zn_7_MT2 and Zn_4_MT2 can only be possible if they share a similar metal configuration. The Cys residues in the α- and β-domains are Zn(II)-protected, and thus they are not easily accessible to IAM. Upon increase of IAM concentration, there is Zn(II) dissociation and subsequently Cys modification in both Zn_7_MT2 and Zn_4_MT2 systems (Figure 2B). In contrast, as expected, Cd_7_MT2 and Cd_4_MT2 behave differently, since the four Cd(II) ions are strongly bound to one domain and they do not redistribute within both domains (Figure 2C). Migration-induced alkylation is a major point of concern in chemical labeling, and thus the reaction of Zn_4_MT2 with IAM may induce metal migration to energetically more favorable binding sites. However, IAM did not induce metal ion migration in this study, and it was used to map regions where the metal binds. In this respect, native top-down MS was performed on free-label apoMT2, Zn_4_MT2, and Cd_4_MT2 proteins activated by collision-induced dissociation (CID) to gain insight into the region involved in the protein–metal interactions. The 5+ charge state for apoMT2, Cd_4_MT2, and Zn_4_MT was isolated and subjected to fragmentation using CID (Figure 2D–F). However, metal/ligand dissociation or migration could happen before backbone fragmentation using CID.^69^ A key factor to maintain the ligand–protein interactions is the stability of the gas-phase complex.^69^ The affinity of the first four Zn(II) ions bound to MT2 is found to be similar in both the gas and solution phases, with an apparent binding constant *K*_b_ of ∼10^11^ M^–1^ for each Zn(II) ion (Figure S4A,B). The three remaining Zn(II) binding sites are strengthened in a vacuum with a resulting stability constant *K*_b_ ∼ 10^11^ M^–1^. In consequence, metal ion dissociation upon CID activation is unlikely to occur under controlled conditions. This effect has been previously reported and attributed to the differences in the dielectric permittivity between water and a vacuum that increases the electrostatic interactions stabilizing the gas-phase complex.^70^ Noncovalent interactions can be preserved in low solution binding sites (*K*_d_ 10^–3^ M), as previously demonstrated.^71^ To avoid undesired effects involving nonvolatile salts during the ESI process, such as signal suppression, native MS usually requires exchanging of a pH-buffered solution with a physiological salt concentration with a neutral pH MS-compatible solution (i.e., ammonium acetate or formate). However, these solutions do not constitute a buffer at neutral pH.^44,45^ For example, neutral ammonium acetate provides buffering capabilities at pH 4.75 and 9.25 ± 1, corresponding to the acetic acid and ammonium p*K*_a_s. Therefore, it is likely that proteins undergo acidification in the ESI plume. The pH drop can be less dramatic when high concentrations of ammonium acetate (∼100 mM) are used. Under these conditions, the pH should only drop to 6.5 units.^45^ These solutions do not contain any salt that could stabilize noncovalent interactions.^44^ One solution to solve such limitation is the use of nanoemitters.^42^ In the case of human MT-2, each of the seven Zn(II) ions do not bind with the same affinity, and the last one binds with nanomolar affinity.^12^ Previous reports have shown that such low affinity sites can be strengthened in a vacuum.^47,51^ Lower binding affinity of a zinc finger CP1 for Zn(II) was found in MS experiments compared with the value found in solution.^39^ Another factor that can alter the *K*_d_s are the so-called “response factors” which refers to the different ionization state of the free and bound protein.^72^ In this case, we expect that all the Zn_0–7_MT2 species ionize similarly since Zn(II) is a small cation which will unlikely alter the ionization properties of the protein. Lastly, supermetallization of peptides and proteins has been observed during ESI, suggesting that the ESI conditions should be carefully monitored.^73^ In agreement with previous reports,^47,36^ the stability constant has been estimated to be *K*_b_ ∼ 10^11^ M^–1^ in the gas phase for the last three Zn(II) binding events. These values are slightly overestimated compared to the solution, which is likely a result of the above-mentioned observations.

For the apoMT2 system, *b*- and *y*-type fragment ions derived from both α- and β-domains are identified in the spectra (Figure 2D). Addition of 4 Cd(II) equiv to the apoMT2 system yields Cd_4_MT2 (Figure S5). CID fragmentation of the 5+ charge state shows metal-free *b*-type fragment ions from the backbone dissociation of the β-domain and two metal-free fragments, *y*_10_^+^ and *y*_24_^2+^, the latter associated with the proline effect.^74^ Fragments of *y*-type and *b*-type that retained four Cd(II) ions were also identified. Observation of a metal-bound ion fragment provides some evidence that at least one Cys residue coordinates a metal ion, and the presence of a metal-free fragment indicates that the region weakly interacts with the metal ion. Thus, the presence of [*b*_60_ + 4Cd]^5+^ indicates that the entire α-domain is involved in binding four Cd(II) ions (Figure 2E). It cannot be concluded from the fragmentation that the β-domain does not participate in metal coordination since the lower fragment found was [*y*_51_ + 4Cd]^5+^. Upon addition of 4 Zn(II) equiv to the apoMT2 form, the mass spectra reveal less cooperative binding in the presence of multiple Zn(II)-depleted states (Figure S5). Generally, the CID fragmentation spectra were similar for the Zn_4_MT2 and Cd_4_MT2 systems, sharing many metal-free and *y*-type fragment ions. However, [*b*_38_ + 3Zn]^4+^, [*b*_29_ + 3Zn]^3+^, and [*b*_42_ + Zn]^4+^ fragment ions were identified in the MS/MS spectra (Figure 2F), which demonstrates the localization of the four Zn(II) ions in Zn_4_MT2. The CID spectra strongly suggest that Zn(II) ions weakly interact with the N-terminal and that they are coordinated to residues in both the α- and β-domains. The binding regions identified using a top-down MS approach are consistent with the results from the chemical labeling approach.

### SMD to Investigate the Unfolding Dynamics of Zn_7_MT2 and Cd_7_MT2: The Role of Metal Clusters in Protein Folding

**Figure 3:**
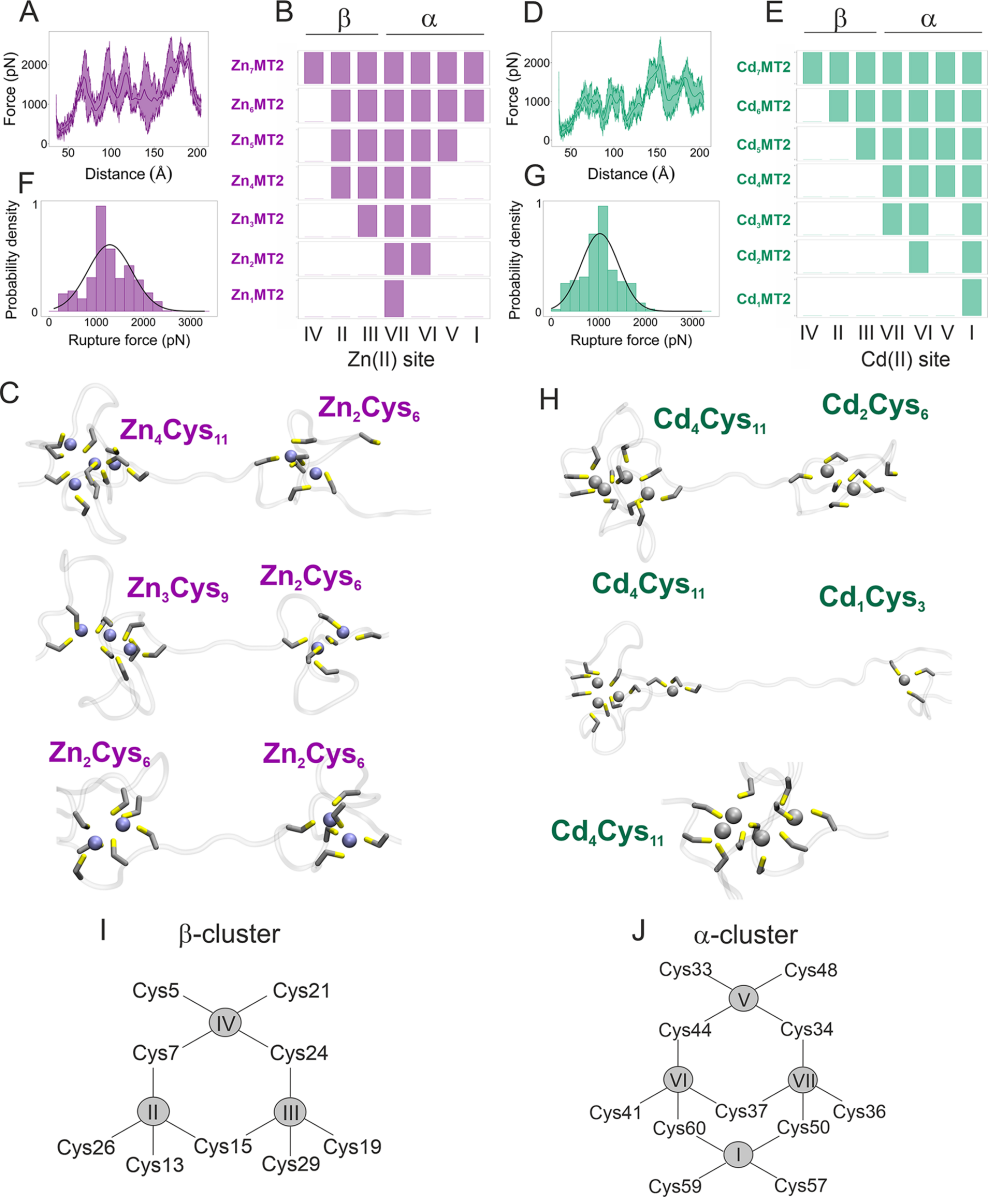
SMD simulations involving Zn_7_MT2 and Cd_7_MT2 with the C-terminal fixed. Force–extension curves calculated from SMD simulations for the most populated pathways for (A) Zn_7_MT2 and (D) Cd_7_MT2. The solid green line refers to the mean, and the shaded area denotes the standard error calculated. A force constant of *k* = 3 kcal/mol (pN/Å) was applied on the N-terminus with a constant velocity of 100 Å/ns. Order of the stepwise Zn(II) dissociation for the most common found unfolding pathway for (B) Zn_7_MT2 and (E) Cd_7_MT2. The bars show the binding/unbinding of the metal ion for each metal-loaded species. The metal ions in the α- and β-domains are shown in (I) and (J). Representative conformations when 6, 5, and 4 metal ions are bound to the protein in the presence of (C) Zn(II) or (H) Cd(II) ions. The metal sites for every configuration can be elucidated from (B) and (E). Zn(II) and Cd(II) are represented by a purple and gray spheres, respectively, and the S atoms are shown in yellow. Rupture force histogram fitted to a unimodal Gaussian distribution for the most populated pathways for (F) Zn_7_MT2 and (G) Cd_7_MT2; an average rupture force of 1252 ± 438 pN and 1032 ± 481 pN was determined, respectively. Schematic representation of the β- (I) and α-clusters (J). Purple and gray circles refer to Zn(II) and Cd(II) ions, respectively. The metal ions are specified by roman numerals and correspond to the ^113^Cd resonances according to their decreasing chemical shifts in the NMR spectra.

To investigate the protein unfolding mechanism, 100 SMD runs were performed at constant velocity (100 Å·ns^–1^) using the Zn_7_MT2 and Cd_7_MT2 systems. The force–extension curves showed a sawtooth-like pattern with multiple unfolding steps,^75^ where each force peak is concomitant with the dissociation of Zn–S bonds and rupture of Zn_*x*_Cys_*y*_ clusters, and therefore it is a direct measure of the stability of the interaction (Figure 3A). Three different unbinding pathways, A, B and C, were identified (Figure S6). These pathways share some similarities. For example, the first Zn(II) ion dissociates from the β-domain in all of pathways (position IV, Figure 3).

This event is found in 70% of the SMD runs (Figure 3B and Table 3). In the A (Figure 3B) and C (Figure S6F) pathway, Zn(II) dissociates from the β-Zn_3_Cys_9_ cluster following a noncooperative mechanism, in agreement with the multistep unfolding that had been previously experimentally observed for a Cd_3_Cys_9_ cluster.^75^ In the Zn_7_MT2 protein, the β-Zn_3_Cys_9_ cluster breaks first, forming a β-Zn_2_Cys_6_ cluster, and subsequently the α-Zn_4_Cys_11_ is disrupted, yielding an α-Zn_3_Cys_9_ cluster and finally an α-Zn_2_Cys_6_ cluster (Figure 3B,C and Table 3). Zn_4_MT2 was found in 60% of the pathways. In agreement with the MS data recorded, two Zn(II) ions are bound in each domain, herein identified to form a Zn_2_Cys_6_ cluster. To strengthen our conclusions, we found that this Zn_4_MT2 conformation is independent of the tethering geometry by performing SMD simulations with the N-terminal fixed. The final unfolding steps proceed first by Zn(II) dissociation and unfolding of the β-domain, followed by unfolding of the α-domain (Figure 3B,C and Table 3). In the B pathway, Zn(II) is first dissociated from the β-domain and then from the α-domain (Figure S6). As a control, similar SMD simulations were performed for the isolated β-Zn_3_Cys_9_MT2 domain from the α,β-Zn_7_MT2 protein. Nine different unfolding events were recorded, one of which is repeated 43 times. The β-Zn_3_Cys_9_ cluster breaks to produce the β-Zn_2_Cys_6_ cluster that is subsequently unfolded (Figure S7).

**Table 3:** Comparison of the Domain Location of the Zn(II) Binding Sites for Zn_1–7_MT2 Species Inferred Throughout the Three Methodologies Employed^a^

species	MS	SMD	WT-PBMetaD
Zn_1_MT2	α	1α	1β
Zn_2_MT2	2α	2α	1α + 1β
	1α + 1β		
Zn_3_MT2	1α + 2β	2α + 1β	2α + 1β
	2α + 1β		
Zn_4_MT2	2α + 2β	2α + 2β	2α + 2β
			3α + 1β
Zn_5_MT2	3α + 2β	3α + 2β	3α + 2β
Zn_6_MT2	4α + 2β	4α + 2β	4α + 2β
Zn_7_MT2	4α + 3β	4α + 3β	4α + 3β

a The α and β denote the domain where the Zn(II) is bound. Mass spectrometry refers to the conclusions obtained with the combination of native MS, MALDI-MS, and LC-MS/MS. SMD refers to well-termpered steered MD simulations and WT-PBMetaD to parallel bias metadynamics.

Unfolding of the Cd_7_MT2 protein was studied by performing 100 SMD runs at constant speed. Solution- and gas-phase experimental studies precisely determined that four Cd(II) ions are bound in the α-domain; thus these simulations serve as an additional control. Twelve possible unfolding pathways were identified in the simulated trajectories, and a stable Cd_4_MT2 conformation where four Cd(II) ions are located in the α-domain was obtained. The force–extension profile shows a maximum force peak corresponding to the disruption of the Cd_4_Cys_11_ cluster (Figure 3D). This conformation was found in 97% of the SMD simulations. The first Cd(II) ion dissociated from the β-domain in the most populated pathway (Figure 3E), which is a similar feature observed in the Zn_7_MT2 unfolding pathway. To calculate the average rupture force, histograms were fitted to unimodal Gaussian distributions that yielded similar average forces of 1252 ± 438 pN for Zn_7_MT2 in comparison to 1032 ± 481 pN for Cd_7_MT2 (Figure 3F,G). During the unfolding process, Zn(II) and Cd(II) ions differ in the metal clusters that they form (Figure 3C,H–J), and this is evidenced in the force–extension profiles where all the peaks have similar rupture force for Zn_7_MT2 (Figure 3A) while a maximum force peak is found for Cd_7_MT2 (Figure 3D). In Zn_7_MT2, Zn(II) dissociates in a stepwise manner, whereas in Cd_7_MT2, a stable Cd_4_MT2 intermediate is formed, and the four Cd(II) ions are found in the α-domain (Figure 3I,J). SMD simulations reproduced well the in-solution findings for Zn_4_MT2 and Cd_4_MT2, and therefore these results are consistent with the different reactivities found in the MS experiments. SMD simulations also provide detailed structural information at the molecular level. The complementary SMD simulations performed on the isolated β-domain rendered similar Zn(II) dissociation pathways as those observed for Cd(II) when dissociating from Cd_7_MT2.

### Mechanistic Details of Zn(II) Binding to MT2 at the Molecular Level

**Figure 4:**
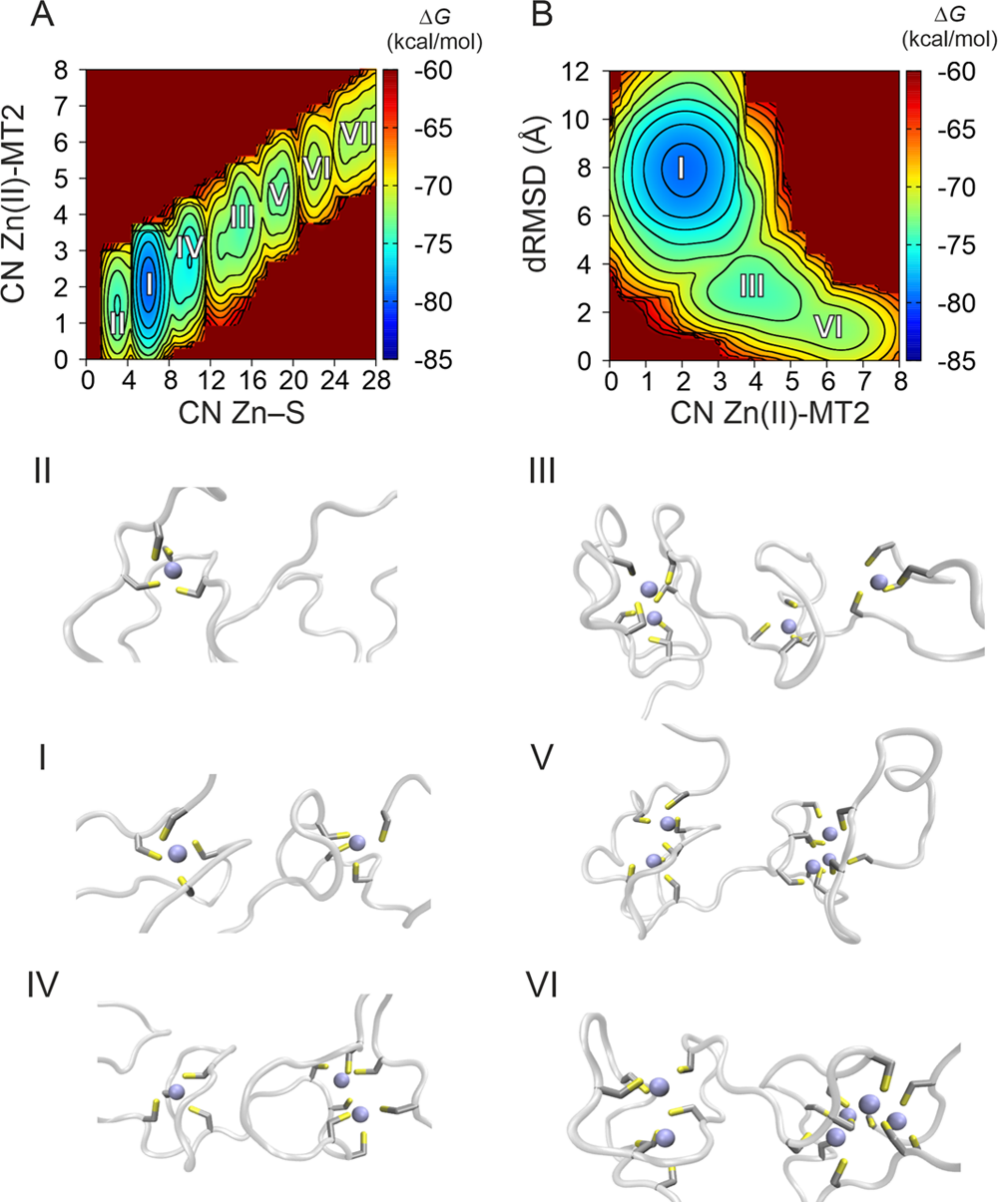
(A) Reweighted FES from WT-PBMetaD simulations of Zn_7_MT2 as a function of the number of contacts between Zn(II) and 20 sulfur atoms from the Cys residues in the protein (CN Zn–S) and the CN between Zn(II) and MT2 (CN Zn(II)-MT2) which measures the total number of Zn(II) ions bound to the protein. (B) Reweighted FES as a function of CN Zn(II)-MT2 and the dRMSD with respect to the native Zn_7_MT2 configuration. The energy is given in kilocalories per mole, and each isoline corresponds to 1.5 kcal/mol. Representative snapshots of the structures corresponding to each energy minimum in the FES are shown and numbered according to their energies. Sulfur atoms and Zn(II) ions are shown in yellow and gray, respectively, and the protein backbone in white licorice representation.

To study the effects of Zn(II) binding on the conformational landscape of MT2 and to provide a quantitative characterization of the Zn(II) binding states, well-tempered parallel bias metadynamics was employed.^66^ To describe both Zn(II) depleted and Zn(II) bound MT2 states, seven CVs were used, each one describing the number of contacts between one of the seven Zn(II) ions present in MT2 and each of the sulfur atoms from the 20 cysteine residues involved. The choice of these CVs allows energetic characterization of the different bound states involved in the different mechanisms described earlier and energetical classification of each pathway (Figure S8). The starting point was a system where each Zn(II) ion is bound to four Cys residues. Subsequently, each Zn(II) was forced to explore another configuration where it is coordinated by a different number of Cys residues and/or water molecules. A multidimensional free energy surface was obtained describing the different loading states of each of the Zn(II) ions considered and the 20 Cys residues present in the protein. Then, the FES of the Zn(II)-MT2 configurations was estimated as a function of two CVs different from those biased by applying an umbrella-sampling-like reweighting algorithm (Figure 4).

The two CVs employed were the Zn–S contact number (CN Zn–S) and the Zn(II)-MT2 contact number (CN Zn(II)-MT2) (Figure 4A). A quantitative estimation of the binding states found as a function of these two CVs is shown in Table S3. Experimentally, the binding affinities of each of the seven Zn(II) binding sites are known, but which affinity corresponds to which Zn(II) binding site is unclear. The free energies obtained from metadynamics could reproduce the trend of the experimental binding affinities: The first four Zn(II) (Zn_1–4_MT2) are bound more strongly than the remaining three Zn(II) (Zn_5–7_MT2) (Table S3).^12^ During the WT-PBMetaD simulations, one Zn(II) binding event is reported in the β-domain (basin II in Figure 4), and a second one (basin I in Figure 4) corresponds to a Zn_2_MT configuration (CN Zn(II)-MT2 = 2, CN Zn–S = 6–8). Two main configurations resulted from a cluster analysis of basin I: (i) one with two ZnCys_4_ sites in the α- and β-domains, respectively, and (ii) a second which is a β-Zn_2_Cys_7_ cluster. A third free-energy minimum ∼7 kcal·mol^–1^ higher than the one observed in basin I was identified which corresponds to a Zn_3_MT2 system where two Zn(II) ions are bound to the α-domain and one Zn(II) ion is bound to the β-domain (basin IV in Figure 4). Two main clusters of stoichiometry Zn_4_MT2 correspond to basin III which is 1 kcal·mol^–1^ lower relative to basin IV. One of these clusters corresponds to a configuration where two Zn(II) ions are bound to each α- and β-domain, forming two α-ZnCys_4_ and one β-Zn_2_Cys_7_ cluster. A second configuration was found with three Zn(II) ions bound to the α-domain and one Zn(II) bound to the β-domain. The configurations found in basin III also support the above-mentioned results, indicating that the α-domain is not fully loaded with four Zn(II) ions in the Zn_4_MT2 complex. The fifth and sixth Zn(II) ions bind to the α-domain (basins V and VI in Figure 4). A representative snapshot of energy minima VI shows a configuration with a Zn_4_αZn_2_βMT2 stoichiometry, confirming that the last and weakest Zn(II) ion binds to the β-domain (basin VI in Figure 4). The coordination dynamics for MT2 were observed when the FES was estimated as a function of the dRMSD with respect to the folded Zn_7_MT2 configuration and CN Zn(II)-MT2 (Figure 4B). A metal-coupled folding effect was observed with three energy minima. This result is in agreement with previous reports where three states of Zn(II)-loaded MT were studied by voltammetric and chemometric analysis.^14^

All configurations elucidated from chemical labeling-MS, SMD and WT-PBMetaD simulations are reported in Table 3. Overall, there is good agreement among these different methods, albeit with some differences regarding configurations Zn_1_MT2 and Zn_2_MT2 obtained from SMD simulations.

### Role of Solvent and Intermolecular Interactions in the Stability of Partially Zn(II)-Depleted MT2 Species

Generally, mediation of water molecules in the binding of ligands to proteins has been extensively reported in the literature.^76−78^ In particular, Zn(II)-aquo complexes, limited here to the hexa-aquo complex [Zn(H_2_O)_6_]^2+^, exchange their water molecules when binding to protein coordinating residues such as Cys residues.^79^ Experimentally, one may determine the binding affinity of Zn(II) to the protein,^80^ but it is rather difficult to study in detail the relative free energy of the different binding states. The availability of oxygen, nitrogen, and sulfur donors modulates the coordination number and geometries of Zn(II) in proteins. The most common coordination numbers of Zn(II) binding sites are 4, 5, and 6.^61,79^ Solvation effects on Zn(II) sites have been studied in isolated β- or α-domains.^81^ The different energy tendencies for Zn(II) dissociation in the presence and absence of solvent molecules and depending on the protein environment have been reported;^81^ the role of the solvent dictated a slightly preferential Zn(II) dissociation from the β-domain. However, it has experimentally been shown that the cumulative properties of each individual domain do not explain the structural properties of the full-length protein.^32,82^ For example, the nanomolar affinity of Zn(II) sites is only found in the two-domain structure, indirectly suggesting that intermolecular interactions and/or changes in the solvation effects are critical for the native function of the protein. Researchers have used isolated protein domains in numerous functional studies, especially when Cd(II) is used as a Zn(II) isostructural replacement.^83−85^ In order to clarify the validity of this approach, the solvation and structural properties of partially Zn(II)-depleted complexes were characterized. The energetic contribution of water solvent to each of the seven Zn(II) binding states was obtained by computing the FES of Zn(II) binding to MT2, using a reweighting technique and constructed as a function of two CVs: (i) the CN between a particular Zn(II) ion and the Cys residues from the protein (CN_Zn–S_) and (ii) the CN between a Zn(II) ion and the oxygen atoms of water molecules (CN_Zn–O_). The starting configuration was Zn_7_MT2, where each Zn(II) ion is coordinated by four Cys residues and experiences a change in the coordination number and geometry from tetrahedral (CN_Zn–S_ = 4) to octahedral (CN_Zn–O_ = 6), which are the most stable coordination numbers (Figure S9).^76^

A variety of binding states are unveiled from the FES computed for the Zn(II)-MT2 system, with distinct contributions from water to the metal sites (Table S4). Five different Zn(II) global minima sites were found, corresponding to site I (CN_Zn–S_ = 2, CN_Zn–O_ = 3), site V (CN_Zn–S_ = 1, CN_Zn–O_ = 4), site VI (CN_Zn–S_ = 1, CN_Zn–O_ = 5), sites VII and II (CN_Zn–S_ = 3, CN_Zn–O_ = 3), and sites III and IV (CN_Zn–S_ = 2, CN_Zn–O_ = 4) (Figure S9). As expected, Zn(II) binding sites are different and they are stabilized by different numbers of water molecules. For instance, Zn(II) unbound states at site II are highly unfavorable in comparison to other binding sites (Figure S9). This is attributed to the higher binding affinity of Zn(II) for site II and in agreement with the configuration found in basin II (Figure 4). The free energies of the most likely bound states for each Zn(II) site are reported in Table S4.

It is well documented that there exists a trade-off between electrostatic interactions and structural flexibility for intrinsically disordered proteins^86^ where the formation and dissolution of salt bridges and the interchange between charged atom pairs modulate their conformations. Hydrogen bonds, salt bridges, and electrostatic interactions were analyzed in the structures identified in the main basins of the FES (Figure 4). No salt bridges or other structural elements that stabilize the interdomain α–β interaction were found (Table S5). The Lys30 residue in the linker region was observed to electrostatically stabilize the β-domain via an interaction with the negatively charged Cys19 residue in the Zn_5–7_MT2 complex. The electrostatic interactions between a positively charged atom and a negatively charged Cys residue would downshift the Cys19 p*K*_a_, and therefore this effect raises their reactivity toward Zn(II), in this particular case, site III.^87^ In order to test the differential Cys reactivity, the free energy profile for the Zn–S bond dissociation was determined with WT-MetaD using the Zn–S distance as CV.

**Figure 5:**
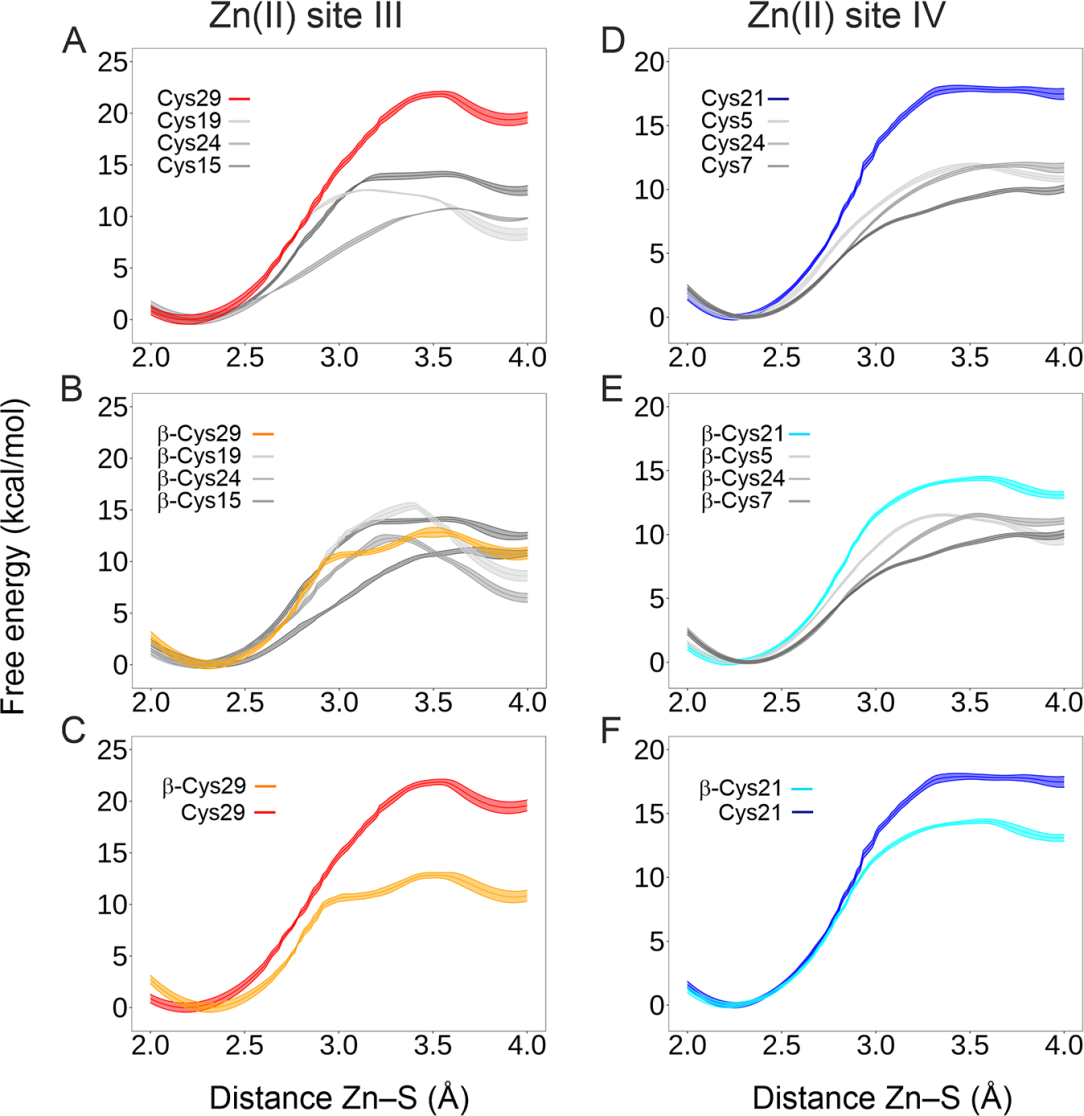
Estimated free energy profiles for the Zn(II)-Cys(S) dissociation for the Zn(II) sites III (A–C) and IV (D–F) obtained from WT-MetaD simulations. The distance between the Zn(II) ion and the S atom of each Cys residue involved in the Zn(II) sites III and IV was used as CV. The solid line refers to the mean, and the shaded area denotes the free energy error calculated by block analysis. The Zn_7_MT2 (A and D) and isolated β-Zn_3_MT2 domain (B and E) systems were used. The hyper-reactive Cys29 and Cys21 residues identified (A and D) are only present in the Zn_7_MT2 system and not in the β-Zn_3_MT2 domain (C and F).

The Lys30-Cys19 interaction had to result in a metastable Zn–S(Cys19) bond. However, the results clearly differed (Figure 5A). Surprisingly, Cys29 but not Cys19 is found to be more thermodynamically stable with respect to the rest of Cys residues at site III (Figure 5A), although this character was diminished in the absence of the α-domain (Figure 5B,C). Analogously, WT-MetaD simulations were conducted for all the Zn(II) sites. The Zn–S(Cys21) bond at site IV was also found to exhibit hyper-reactivity (Figure 5D). Once more, its reactivity was abolished in the absence of the α-domain (Figure 5E,F). To investigate this further, WT-MetaD was performed for Cd(II) sites in Cd_7_MT2. Remarkably, the Cd–S(Cys29) and Cd–S(Cys21) bonds did not show any hyper-reactive character (Figure S10).

Indeed, the major stabilizing factor for each individual domain in MTs is the metal–S bonds.^72^ However, considering only the metal–S bonds could not explain the protein reactivity or the underlying function, as otherwise the properties of the individual domains could reproduce the properties of the two-domain protein.^80^ Taken together, the data presented in this study demonstrate that the α-domain regulates the reactivity of a number of Cys residues in the β-domain. Therefore, the use of isolated domains in biophysical studies of Zn(II)-MTs should be discouraged. Overall, logically, no single residue can explain the reactivity character of the whole protein, which is a result of cumulative factors. The conformational constraint imposed by the two domains of the protein appears to be a major factor explaining the nature of the protein, in addition to the inherent nature of the metal; Zn(II) and Cd(II) ions provide different and unique chemistry that is reflected in the structure and reactivity of MTs.

## Conclusions

Despite more than 60 years of investigation focused on MTs, their native functions are not yet fully understood. A milestone in mammalian MTs research was the discovery of the concentrations of cellular free Zn(II) and their fluctuations as well as the different affinities of Zn(II) ions for MTs. Considering that cellular free Zn(II) concentrations overlap with Zn(II) dissociation constants of MTs, it became clear why naturally occurring MTs are not fully saturated under physiological conditions and why their speciation is correlated with dynamic zinc homeostasis. The importance of partially depleted or saturated species is clearly noticeable in the cellular zinc buffering mechanism. Both the spatial organization and the mechanism of formation of these biologically relevant MT forms have remained elusive due to their lack of secondary elements, the spectroscopic silent character of Zn(II), and the highly dynamic character of Zn(II)-binding sites. A number of studies have employed Cd(II) as an isostructural Zn(II) replacement because Cd(II) provides well-defined spectroscopic signals. However, there are striking differences between these metal ions which result in the formation of different metal intermediates and binding affinities. Therefore, complementary methods to address subtle but highly relevant biologically differences between very similar partially Zn(II)-depleted MT2 protein states are crucial. In an effort to better understand the structure of Zn(II)-MT2 complexes, a combined strategy using MS and MD approaches was employed. The comprehensive integration of MS and MD methods has provided new insights into the structural organization, the mechanostability of Zn(II) in partially and fully loaded states, and the binding/dissociation mechanism to/from MT2. Native ESI-MS, chemical labeling, and bottom-up, top-down MS and MD simulations demonstrate that Zn(II) ions in the Zn _1–4_MT2 complexes are bound to both the α- and β-domains, unlike Cd(II) or Cu(I) ions that bind exclusively to either the α-domain or the β-domain forming α-Cd_4_MT2 or β-Cu_4_MT2 clusters. Steered MD and WT-PBMetaD simulations revealed the structural features of Zn_4_MT2, with α-Zn_2_Cys_6_ and β-Zn_2_Cys_6_ configurations. The Zn_5_MT2 complex possesses three Zn(II) ions bound in the α-domain and two Zn(II) ions in the β-domain. A sixth Zn(II) ion binds to the α-domain rendering the Zn_6_MT2 system. The first three metal dissociation events do not correspond to the three binding sites in the β-domain, and therefore it can be explained why the isolated β-domain cannot mimic these Zn(II) sites. Zn_1–6_MT2 complexes are characterized by different numbers of water molecules and solvation features during Zn(II) (un)binding events. Studying their structural properties revealed an electrostatic interaction between Lys30 and Cys19 in the Zn_5–7_MT2 complexes, which should downshift the Cys19 p*K*_a_ and in turn increase their reactivity toward Zn(II). This hypothesis was tested via WT-MetaD simulations, and, surprisingly, the Cys29 and Cys21 residues but not Cys19 in the β-domain showed distinct thermodynamic stability that was abolished in the absence of the α-domain. Together, these results demonstrate that the conformational restraint imposed by the two-protein domains modulates the Zn(II) coordination properties. The thermodynamic and structural information collected in this study provides a novel molecular perspective illustrating how the structural landscape of MT2, and likely other MTs, shapes its function as one of the major cellular Zn(II) buffering systems in the cell. This study highlights an example of how the integration of MS and MD simulations can be used for the structural and thermodynamic characterization of intrinsically disordered proteins that bind transition-metal ions.

## Supporting Information Available

Materials; expression and purification of proteins; Figures S1–S9 (PDF)
