# Supplementary material for: An Integrated Mass Spectrometry and Molecular Dynamics Simulations Approach Reveals the Spatial Organization Impact of Metal-Binding Sites on the Stability of Metal-Depleted Metallothionein-2 Species
Source: J Am Chem Soc. 2021 Sep 3;143(40):16486–501. doi: 10.1021/jacs.1c05495 (PMC8517974; doi:10.1021/jacs.1c05495)
Supplement: Supplementary file 1 — ja1c05495_si_001.pdf [file ja1c05495_si_001.pdf]

Supporting information

An integrated mass spectrometry and MD simulations approach reveals the spatial organization impact of metal-binding sites on the stability of metal-depleted metallothionein-2 species

Manuel David Peris-Díaz[a], Roman Guran[b,c], C. Domene[d,e], Vivian de los Rios[f], Ondrej Zitka[b,c], Vojtech Adam[b,c], Artur Krężel[a*]

[a] *Department of Chemical Biology, Faculty of Biotechnology, University of Wrocław, F. Joliot-Curie 14a, 50-383 Wrocław, Poland*
[b] *Department of Chemistry and Biochemistry, Mendel University in Brno, Zemedelska 1, 613 00 Brno, Czech Republic*
[c] *Central European Institute of Technology, Brno University of Technology, Purkynova 123, 612 00 Brno, Czech Republic*
[d] *Department of Chemistry, University of Bath, Claverton Down, Bath BA2 7AY, UK*
[e] *Department of Chemistry, University of Oxford, Oxford OX1 3TA, UK*
[f] *Functional Proteomics, Department of Cellular and Molecular Medicine and Proteomic Facility, Centro de Investigaciones Biológicas (CIB-CSIC), Ramiro de Maeztu 9, 28040 Madrid, Spain*

TABLE OF CONTENTS

Materials……………………………………………………………..…………………………..S2
Expression and purification of metallothioneins… ……………………………………..…………S2
Figure S1 ………………………………………………………………………………………..S3
Figure S2 ………………………………………………………………………………………..S4
Figure S3 ………………………………………………………………………………………..S5
Figure S4 ………………………………………………………………………………………..S6
Figure S5 ………………………………………………………………………………………..S7
Figure S6 ………………………………………………………………………………………..S8
Figure S7 ………………………………………………………………………………………..S9
Figure S8 ………………………………………………………………………………………..S10
Figure S9 …………………………………………………………………………….………….S11
Figure S10...…………………………………………………………………………….……….S11
Table S1…………………………………………………………………………………..……..S12
Table S2………………………………………………………………………………………....S13
Table S3………………………………………………………………………………………....S13
Table S4…………………………………………………………………………………..……..S14
Table S5…………………………………………………………………………………..……..S16
References ………………………………………………………………………………………S16

EXPERIMENTAL SECTION

Materials. The following reagents were purchased from Sigma-Aldrich: $ZnSO_4 \cdot 7H_2O$, 4-(2-pyridylazo)resorcinol (PAR), $(NH_4)_2CO_3$, tris(hydroxymethyl)aminomethane (Tris base) and 4-(2-hydroxyethyl)-1 piperazineethanesulfonic acid (HEPES), iodoacetamide (IAM), *N*-ethylmaleimide (NEM), tris(2carboxyethyl)phosphine hydrochloride (TCEP), ethylenediamin e-tetraacetic acid (EDTA), proteomics grade trypsin, mass spectrometry grade methanol, and mass spectrometry grade acetonitrile (ACN), 2,5-dihydroxybenzoic acid (DHB) and α-cyano-4-hydroxycinnamic acid (HCCA). The metal-chelating resin Chelex 100 was acquired from Bio-Rad and 98% hydrochloric acid (HCl) was purchased from VWR Chemicals. Tryptone, yeast extract, LB broth, agar, agarose, isopropyl-β-D-1-thiogalactopyranoside (IPTG), and SDS were from Lab Empire, NaCl, NaOH, glycerol, $KH_2PO_4 \cdot H_2O$, K_2HPO_4 from POCH (Gliwice Poland), pTYB21 vector and chitin resin were from New England BioLabs, and 5,5′-dithiobis-(2-nitrobenzoic acid) (DTNB) from TCI Europe N.V. was purchased from Sigma-Aldrich. Dithiothreitol (DTT) was purchased from Iris Biotech GmbH.

Expression and purification of metallothioneins. Expression vectors were transformed into BL21(DE3) *E. coli* cells and cultured in a rich full culture medium (1.1% tryptone, 2.2% yeast extract, 0.45% glycerol, 1.3% K_2HPO_4, 0.38% KH_2PO_4) at 37°C until 0.5 OD_{600}. Cells were induced with 0.1 mM IPTG and incubated overnight at 20°C with vigorous shaking. The next purification steps were conducted at 4°C. Cells were collected by centrifugation (4,000 × *g* for 10 min), resuspended in 50 ml of cold buffer A (20 mM HEPES, pH 8.0, 500 mM NaCl, 1 mM EDTA, 1 mM TCEP), and sonicated for 30 min (1 min "on" and 1 min "off") followed by centrifugation (20,000 × *g* for 15 min). The supernatant was incubated with 20 ml of chitin resin in buffer A and kept overnight with mild shaking. After the incubation, resin was washed 4-5 times with 50 ml of buffer A. Cleavage was induced by adding 100 mM DTT. The resin was incubated for 48 h at room temperature on a rocking bed.[1] Eluted solution was acidified to pH ~ 2.5 with 7% HCl and concentrated using Amicon Ultra-4 Centrifugal Filter Units with a membrane cut-off of 3 kDa (Merck Millipore, USA). It was subsequently purified on a size exclusion chromatography SEC-70 gel filtration column (Bio-Rad) equilibrated with 10 mM HCl.[2] The identity of apo-MT2 protein (thionein) was confirmed by ESI-MS using an API 2000 instrument, Applied Biosystems, USA. The concentration of thiols was determined spectrophotometrically using a DTNB assay.[3] Protein binding capacity was confirmed spectrophotometrically by Zn(II) and Cd(II) titrations.[2] Purified thionein (apo-forms of MT)

were mixed with a 10 molar excess of $ZnSO_4$ under a nitrogen blanket and the pH adjusted to 8.6 with a 1 M solution of Tris base. Samples were concentrated with Amicon Ultra-4 Centrifugal Filter Units with a membrane cut-off of 3 kDa (Merck Millipore, USA). Subsequently, they were purified on a size exclusion chromatography SEC-70 gel filtration column (Bio-Rad) equilibrated with 20 mM Tris-HCl buffer at pH 8.6. Concentrations of thiols and Zn(II) were determined spectrophotometrically using DTNB and PAR assays, respectively.[4]

RESULTS

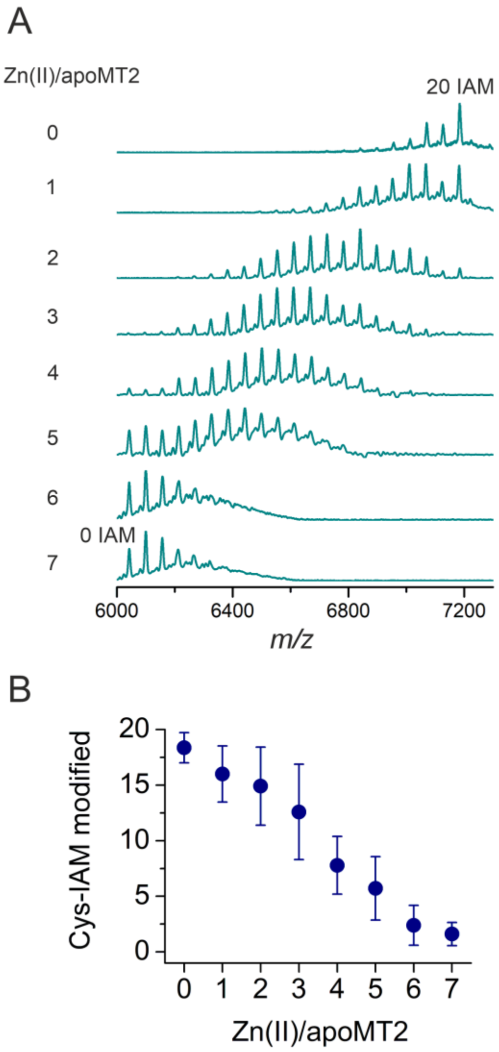

Figure S1. Cysteine profiling for apoMT2 with added 0 to 7 Zn(II) equiv by a single labeling strategy utilizing IAM and monitored with MALDI-MS. (A) MALDI-MS spectra for the stepwise addition of Zn(II) and chemical labeling. (B) Analysis of the MALDI-MS spectra was performed by fitting every mass spectrum to a Gaussian distribution from which the mean and the variance were derived.

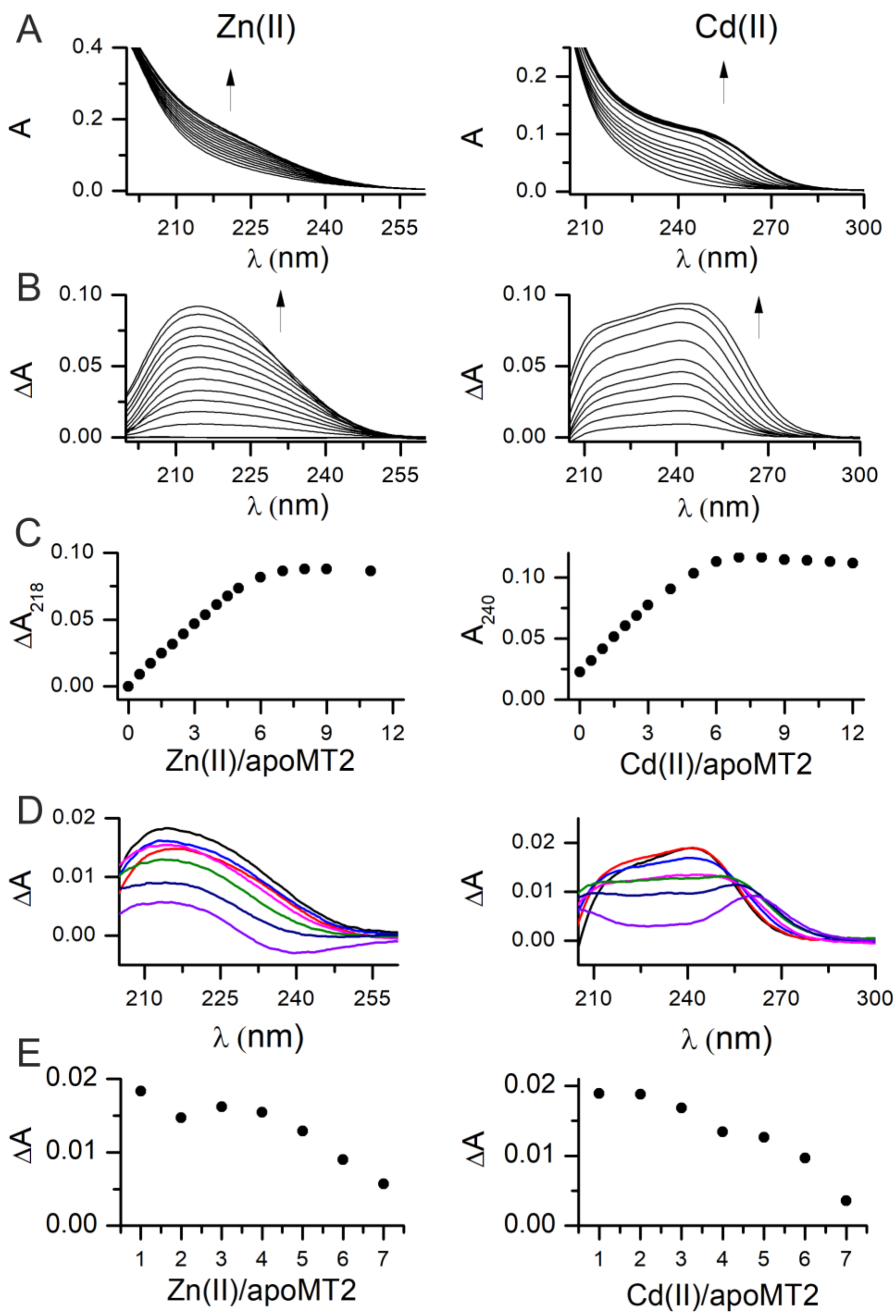

Figure S2. Spectrophotometric titration of 1 μM apoMT2 in 50 mM borate (0.1 M $NaClO_4$, pH 7.4) with Zn(II) (left panel) and Cd(II) (right panel). (A) UV raw spectra. (B) UV-difference spectra obtained by apoMT2 subtractions. (C) Absorbance increase at 218 nm and 240 nm for Cd(II) and Zn(II), respectively. (D) Increment difference absorption spectra obtained by subtracting the successive spectra showed in A. Black, red, blue, magenta, green, navy, and blue indicate spectrum 1 - spectrum 0, 2-1, 3-2, 4-3, 5-4, 6-5 and 7-6, respectively. (E) Absorbance difference between the maximum and the different absorption spectra presented in D. In the case of Cd(II), a bathochromic displacement occurs after the addition 4 Cd(II)/apoMT2 equiv.

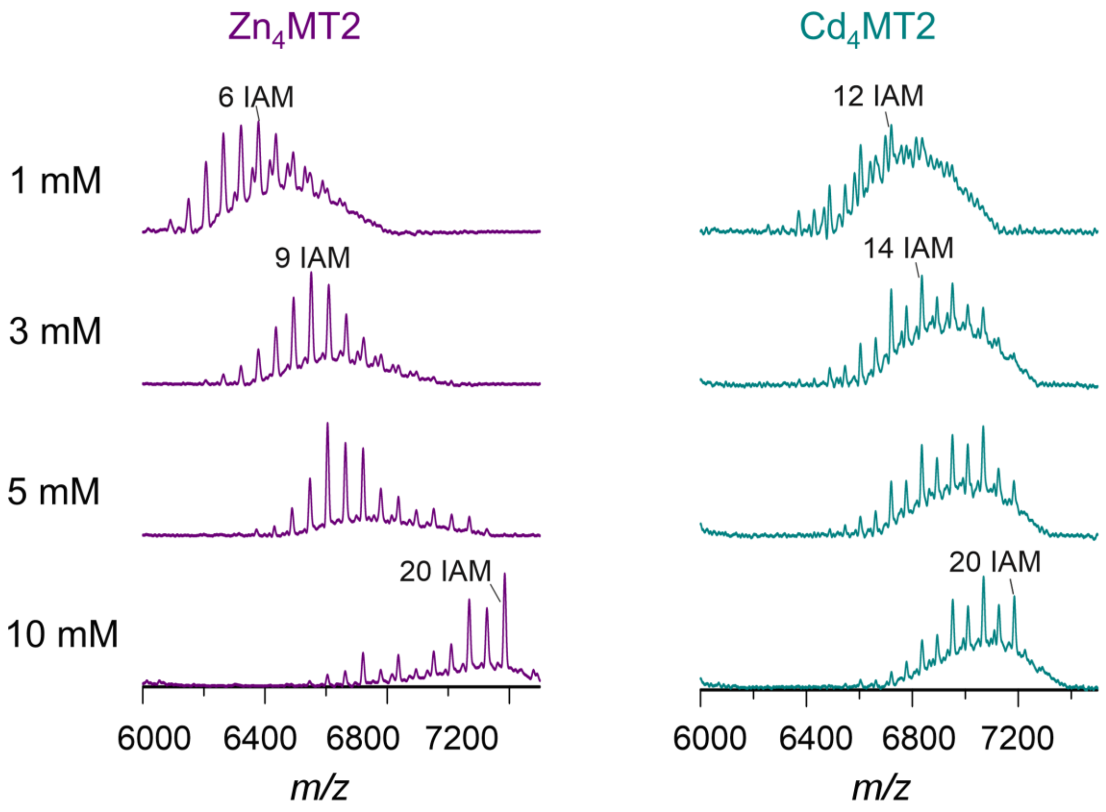

Figure S3. Cysteine profiling for the Zn_4MT2 and Cd_4MT2 systems incubated with increasing IAM concentrations (1 to 10 mM) and monitored by MALDI MS.

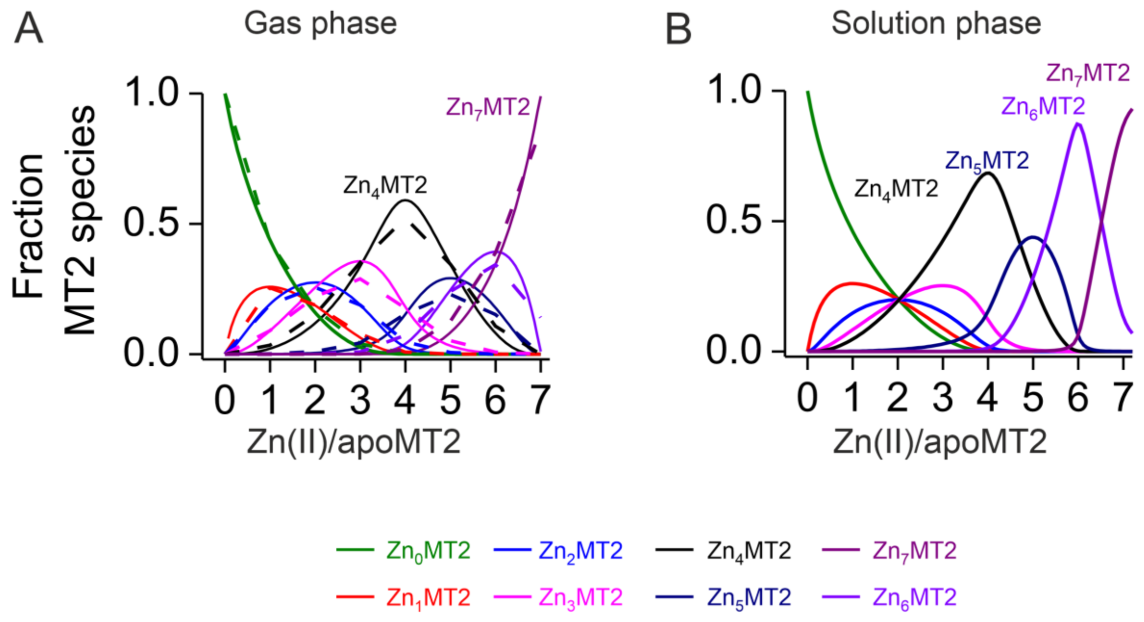

Figure S4. $Zn_{0-7}MT2$ species distribution in gas and solution phase. (A) Speciation plot calculated from the experimental native mass spectra obtained by incubating apoMT2 with 0-4 Zn(II) equiv (dashed lines). The intensities of the signals based on the deconvoluted spectra were used to construct the matrix concentrations. The experimental data were modeled by using the Hyperquad Simulation and Speciation (HySS) software. The simulated data is presented as solid lines.[5] The log β values were: 11.9 (Zn_1MT2), 23.9 (Zn_2MT2), 35.7 (Zn_3MT2), 47.4 (Zn_4MT2), 58.1 (Zn_5MT2), 69 (Zn_6MT2) and 79.6 (Zn_7MT2). The conditions used were 1 μM for apoMT and Zn(II) 0-7 μM. The seven Zn(II) ions are bound with a similar apparent formation constant $K_b \sim 10^{11}$ M^{-1}. (B) Calculated distribution of the $Zn_{0-7}MT2$ species using the published experimental apparent stability constants that were obtained in solution.[1]

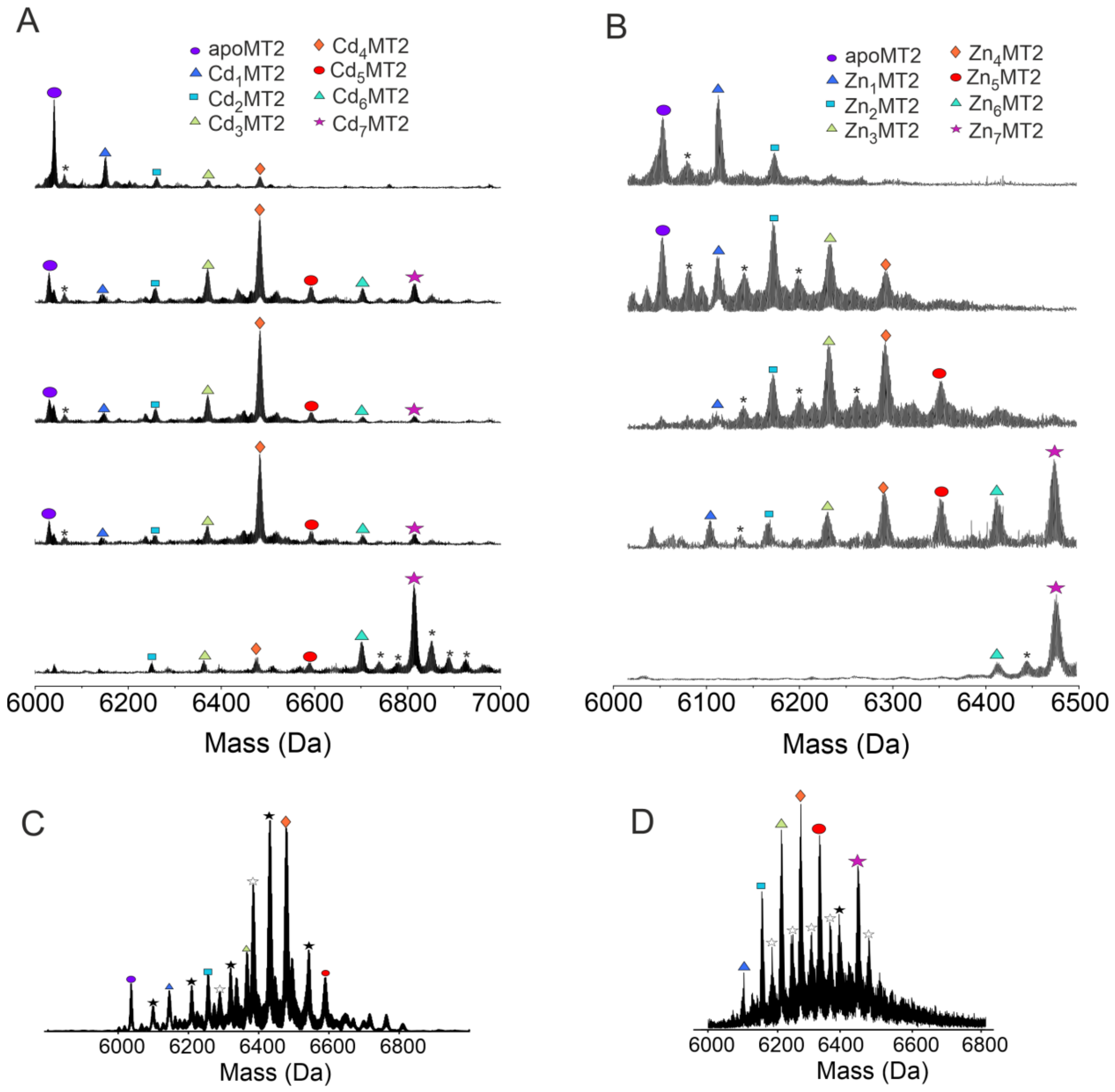

Figure S5. Deconvoluted mass spectrum of apoMT2 (25 μM) incubated with 25-200 μM of (A) Cd(II) and (B) Zn(II) in 50 mM ammonium acetate (pH 7.4). Recorded mass spectrum of apoMT2 after addition of 4 Cd(II) equiv and (C) 4 Zn(II) equiv (D) to apoMT2. Additional peaks at +23 and +60 Da corresponding to sodium and acetate adducts are shown as black and empty asterisks, respectively. The Cd(II) binding mechanism yields a cooperative Cd_4MT2 product, while upon addition of Zn(II), a noncooperative mass distribution with the presence of multiple sequential binding Zn(II)-loaded species is observed. The raw spectra were deconvoluted with the UniDec software.[6]

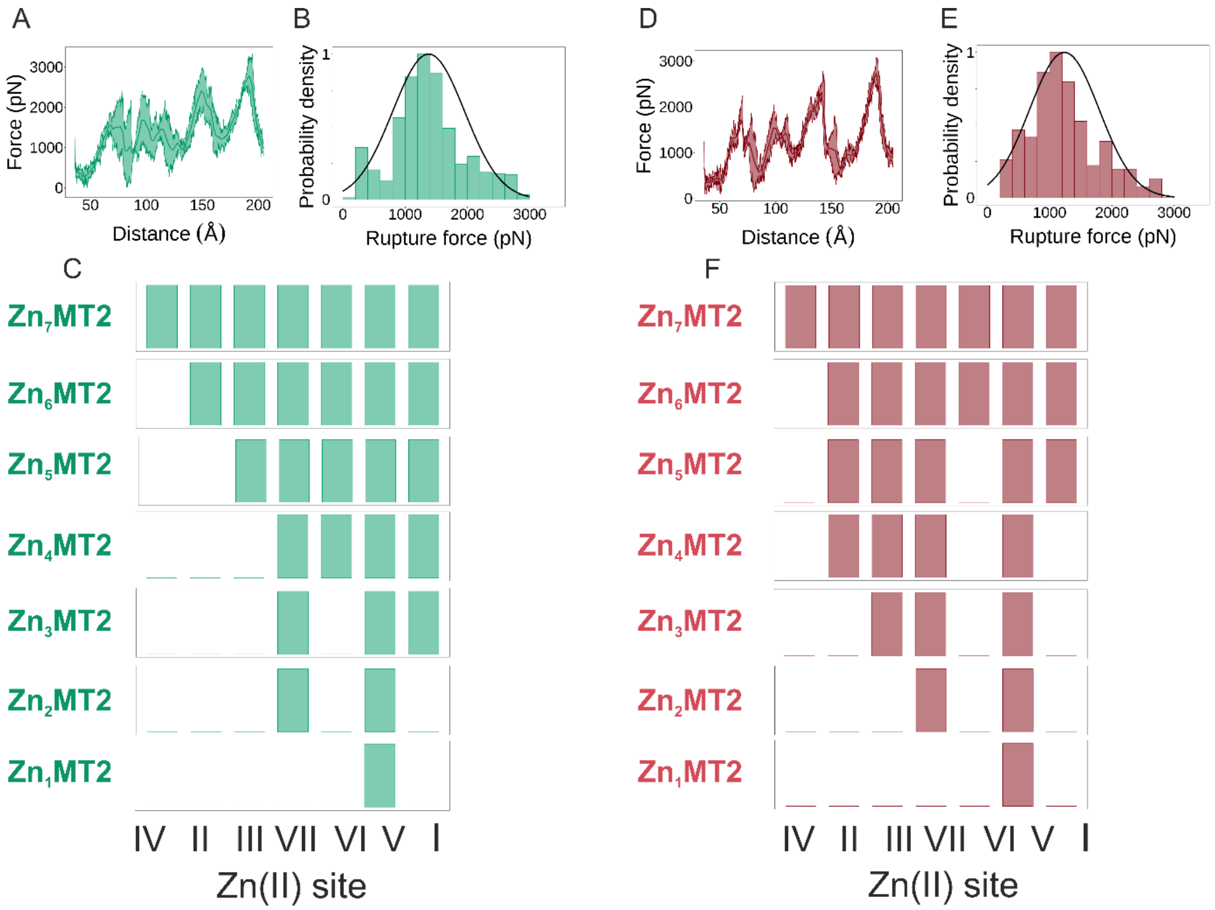

Figure S6. Constant-speed steered molecular dynamics (SMD) simulations of Zn_7MT2 with the C-terminal fixed. Analysis of two out of three (labeled as pathway B and pathway C) unbinding pathways identified in the SMD simulations. (A and D) Force-extension plots where the solid green line refers to the mean and the shaded area denotes the standard error calculated for each of the pathways considered. (B and E) Rupture force histograms were fitted to a unimodal Gaussian distribution to determine an average rupture force of 1377 ± 670 pN and 1237 ± 598 pN, respectively. (C and F) Scheme illustrating the stepwise Zn(II) dissociation mechanism inferred from the computational studies. The binding to or unbinding from a Zn(II) site is represented by a solid bar. The metal ions are specified by roman numerals, which correspond to the metal site in the X-ray structure as presented in Figure 3I-J.

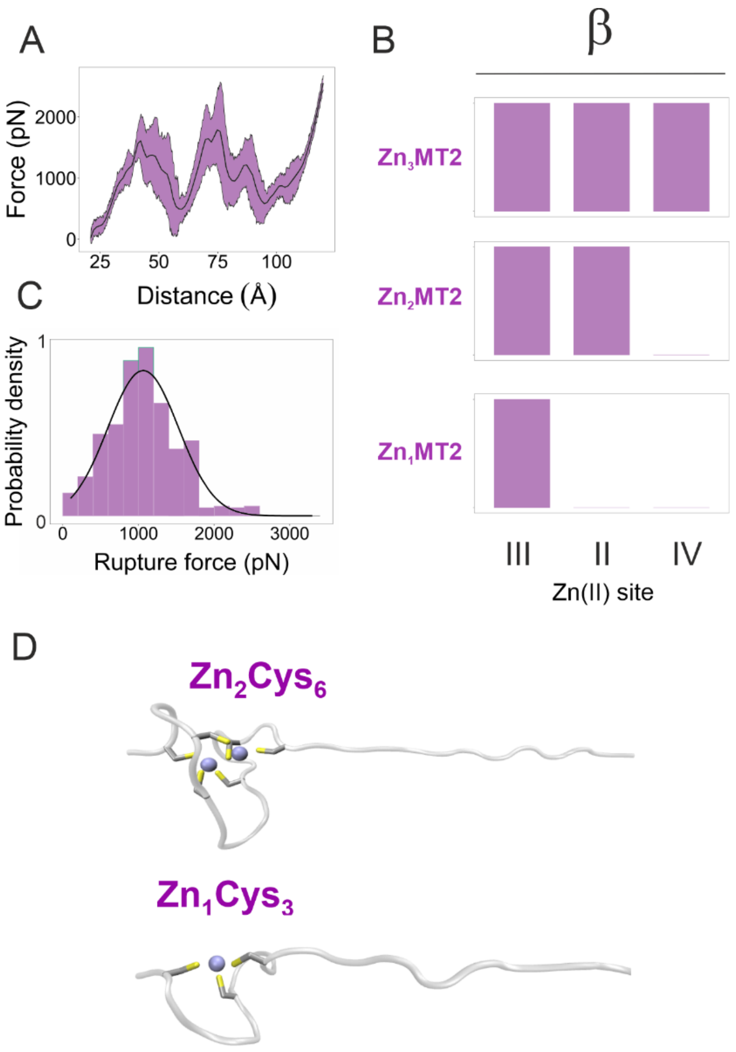

Figure S7. Constant-speed steered molecular dynamics (SMD) simulations of β-Zn_3MT2 with the N-terminal fixed. (A) Force-extension curve for the most common unfolding pathway that was found in 43 out of 100 SMD simulations. The solid green line refers to the mean and the shaded area denotes the standard error calculated from the 43 unbinding pathways. A force constant of $k = 3$ kcal·mol^{-1} (pN/Å) was applied on the N-terminus with a constant velocity of 100 Å·ns^{-1}. (B) Scheme illustrating the stepwise Zn(II) dissociation mechanism inferred from the computational study for the most common unfolding pathway found. (C) Rupture force histogram for the unbinding mechanism identified. The histogram was fitted to a unimodal Gaussian distribution. (D) Representative conformations of the β-Zn_3MT2 system with one and two Zn(II) ions bound to the protein. Zn(II) is represented as a purple sphere, and the S atoms are shown in sticks representations in yellow.

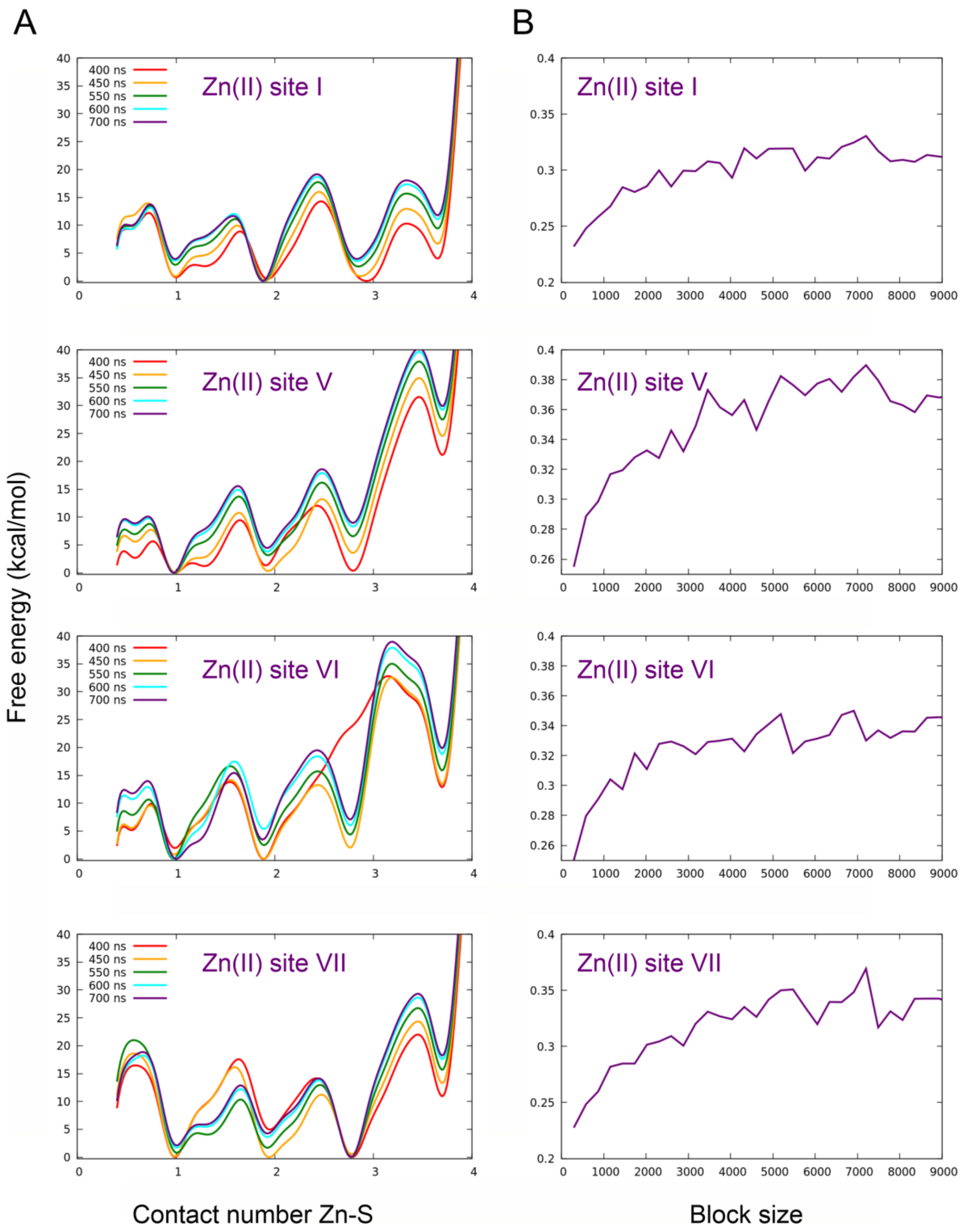

Figure S8. Assessment of the convergence of the PBMetaD simulations. (A) Estimated free energies as a function of the collective variable chosen, which is the contact number between Zn(II) and the sulfur atom of all Cys residues of the protein. (B) Evaluation of the convergence of the estimated free energy from block analysis of the reweighted free energy. The unbiased histograms were obtained using an umbrella sampling-like algorithm where the metadynamics bias potential is taken at the end of the well-tempered parallel-bias metadynamics simulations (0.7 μs).

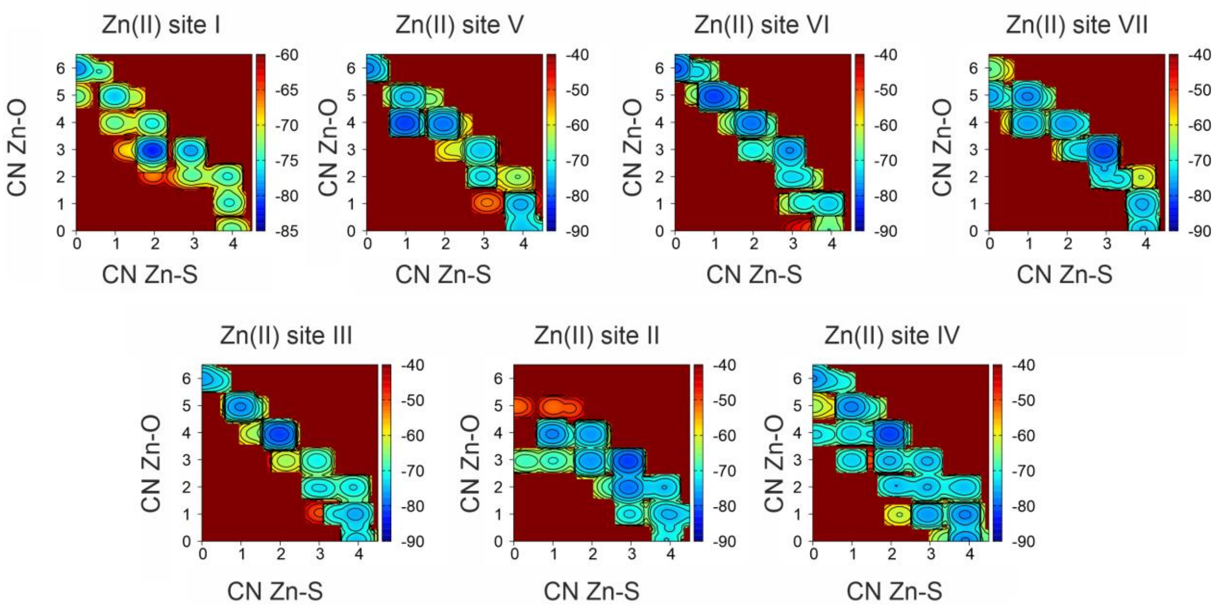

Figure S9. Reweighted free energy surfaces (FES) from the parallel-bias metadynamics (PBMetaD) simulations of Zn_7MT2 as a function of (i) the number of contacts between Zn(II) in the Zn_7MT2 system and the sulfur atoms from each of the twenty Cys residues of the protein (CN Zn–S) and (ii) the number of contacts between Zn(II) in the Zn_7MT2 system and the water oxygen atoms (CN Zn–O). Each FES corresponds to a particular Zn(II) site labeled according to the metal site in the X-ray structure. The energy is given in kcal·mol^{-1}, and each isoline corresponds to 2.5 kcal·mol^{-1}.

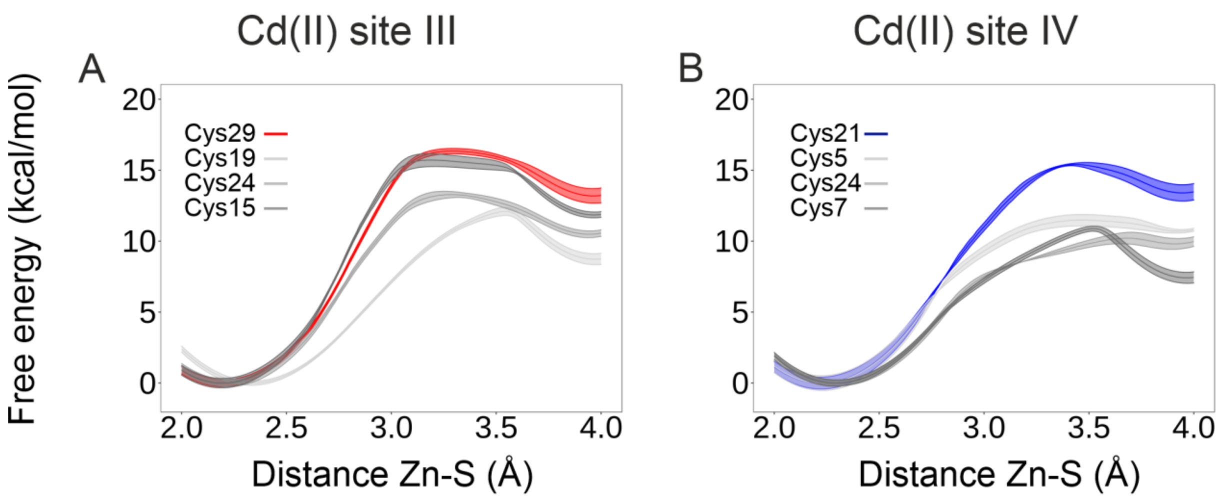

Figure S10. Estimated free energy profiles for the Cd(II)-Cys(S) dissociation for (A) Cd(II) sites III (A) and (B) IV obtained from WT-MetaD simulations. The distance between the Cd(II) ion and the S atom of each Cys residue involved in the Cd(II) sites III and IV was used as CV. The solid line refers to the mean, and the shaded area denotes the free energy error calculated by block analysis.

Table S1. Deconvoluted masses of the apoMT2 system and after addition of *n* Zn(II) equiv. All the systems were labeled with IAM. The mass errors were calculated as the difference between the deconvoluted mass and the calculated mass referred to the monoisotopic mass. The associated MS spectra are shown in Figure 1.

n Zn(II) equiv.	Mass (Da)	Error (Da)	Product ion
0	7179.6	1.9	$Zn_0IAM_{20}MT2$
	7120.3	0.3	$Zn_0IAM_{19}MT2$
	7062.1	1.4	$Zn_0IAM_{18}MT2$
1	7178.4	0.7	$Zn_0IAM_{20}MT2$
	7120.4	0.2	$Zn_0IAM_{19}MT2$
	7072.4	1.9	$Zn_1IAM_{17}MT2$
	6964.0	0.6	$Zn_2IAM_{14}MT2$
2	7183.3	0.1	$Zn_0IAM_{20}MT2$
	7127.3	1.1	$Zn_0IAM_{19}MT2$
	7075.7	0.3	$Zn_1IAM_{17}MT2$
	6967.3	1.5	$Zn_2IAM_{14}MT2$
	6861.3	0.5	$Zn_3IAM_{11}MT2$
3	7179.0	1.4	$Zn_0IAM_{20}MT2$
	7123.1	2.4	$Zn_0IAM_{19}MT2$
	7070.1	0.5	$Zn_1IAM_{17}MT2$
	6963.4	0.3	$Zn_2IAM_{14}MT2$
	6857.6	0.7	$Zn_3IAM_{11}MT2$
	6805.5	1.1	Zn_4IAM_9MT2
	6695.2	2.9	Zn_4IAM_7MT2
	6747.0	2.0	Zn_4IAM_8MT2
	6911.3	2.2	$Zn_3IAM_{12}MT2$
4	6485.8	1.1	Zn_7IAM_0MT2
	6593.8	1.9	Zn_6IAM_3MT2
	6701.8	2.8	Zn_5IAM_6MT2
	6753.3	1.0	Zn_4IAM_8MT2
	6861.3	0.2	$Zn_3IAM_{11}MT2$
	6967.8	0.9	$Zn_2IAM_{14}MT2$
	7073.5	2.5	$Zn_1IAM_{17}MT2$
	7182.7	0.5	$Zn_0IAM_{20}MT2$

Table S2. Difference absorbance values and molar extinction coefficients derived from spectrophotometric titrations of 1 μM apoMT2 in 50 mM borate (0.1 M $NaClO_4$, pH 7.4) with Zn(II) and Cd(II). The ΔA is the difference absorbance obtained by subtracting the successive spectra at the maximum wavelength.

n Zn(II) equiv.	ΔA	Molar absorption coefficients	*n* Cd(II) equiv.	ΔA	Molar absorption coefficients
1	0.0183	18309	1	0.0189	18940
2	0.0147	14700	2	0.0188	18790
3	0.0162	16200	3	0.0168	16850
4	0.0155	15460	4	0.0134	13400
5	0.0129	12890	5	0.0133	13300
6	0.0090	9000	6	0.0114	11400
7	0.0057	5700	7	0.0090	9000

Table S3. Free energies corresponding to the basins shown in Figure 4 of the main manuscript estimated as a function of two CVs: (i) the number of contacts between Zn(II) and the sulfur atoms from the Cys residues of the protein (CN Zn–S) and (ii) the total number of Zn(II) ions bound to MT2 (CN Zn(II)-MT2).

Basin	Specie	CN Zn–S	CN Zn(II)-MT2	Δ*G* (kcal·mol^{-1})	Δ*G* error (kcal·mol^{-1})
II	Zn_1MT2	4	1	-78.9	0.3
I	Zn_2MT2	6-8	2	-80.9	0.4
IV	Zn_3MT2	12	3	-73.5	0.6
III	Zn_4MT2	15	4	-74.7	0.5
V	Zn_5MT2	20	5	-74.1	0.6
VI	Zn_6MT2	25	6	-72.5	0.6
VII	Zn_7MT2	28	7	-71.9	0.5

Table S4. Free energies for the different binding states of each of the seven Zn(II) sites from the reweighted free energy surfaces obtained from the PBMetaD simulations of Zn_7MT2. The free energies were calculated as a function of (i) the number of contacts (CN) between any Zn(II) ion and all the twenty Cys residues in the protein (CN Zn–S) and (ii) the number of contacts between Zn(II) bound to the Zn_7MT2 system and water oxygen atoms (CN Zn–O).

Zn(II) site	CN			Free Energy (kcal·mol^{-1})	Free Energy Error (kcal·mol^{-1})
	Zn–S	Zn–O	Zn–S + Zn–O		
I	4	0	4	-73.9	0.5
	4	1	5	-75.5	0.6
	4	2	6	-75.4	0.6
	3	2	5	-74.1	0.5
	3	3	6	-78.4	0.6
	2	3	5	-81.7	0.6
	2	4	6	-75.9	0.5
	1	4	5	-73.9	0.6
	1	5	6	-77.4	0.3
	0	6	6	-75.2	0.6
V	4	0	4	-74.7	0.3
	4	1	5	-75.9	0.6
	3	2	5	-73.4	0.6
	3	3	6	-74.5	0.6
	2	4	6	-79.2	0.4
	1	4	5	-81.7	0.6
	1	5	6	-75.8	0.6
	0	6	6	-75.7	0.6
VI	4	1	5	-74.2	0.6
	3	2	5	-73.7	0.6
	3	3	6	-77.8	0.6
	2	4	6	-79.1	0.6
	1	5	6	-81.6	0.6
	0	6	6	-81.1	0.6
VII	4	0	4	-75.2	0.5
	4	1	5	-75.8	0.3
	3	3	6	-81.7	0.3
	2	4	6	-77.0	0.6
	1	4	5	-76.8	0.6
	1	5	6	-78.0	0.6
	0	5	6	-66.1	0.6
III	4	0	4	-74.3	0.5
	4	1	5	-76.2	0.3
	4	2	6	-72.8	0.4
	3	2	5	-73.0	0.5
	3	3	6	-71.7	0.4
	2	4	6	-81.7	0.6
	1	5	6	-78.0	0.5
	0	6	6	-76.2	0.6
II	4	1	5	-75.7	0.4
	3	1	4	-73.1	0.6

	3	2	5	-79.1	0.6
	3	3	6	-81.7	0.6
	2	3	5	-77.4	0.6
	2	4	6	-77.2	0.4
	1	4	5	-78.3	0.6
	1	5	6	-52.2	0.6
	0	5	5	-52.0	0.6
IV	4	0	4	-77.8	0.6
	4	1	5	-78.1	0.6
	3	2	5	-75.3	0.2
	3	1	4	-75.6	0.6
	2	3	5	-75.7	0.1
	2	4	5	-81.7	0.6
	1	5	6	-78.4	0.5
	0	6	6	-76.3	0.6

Table S5. Intermolecular interactions in the partially Zn(II)-loaded MT2 states. The salt bridges, H-bonds, and charged atom pairs were analyzed in the basins identified in the FES presented in Figure 4. Salt bridges and charged atom pairs were computed with an oxygen-angle cut-off of 20°. M and S refer to 'main' and 'side' chains.

Basin	Zn(II) location	Specie	Salt-bridge	H-bond (donor-acceptor)	Charged atom pairs
II	α	Zn_1MT2	Asp55-Lys30	Lys25(S)-Met1(M)<br>Ala8(M)-Ser18(M)<br>Cys33(M)-Lys56(M)	-
I	α + β	Zn_2MT2	Asp10-Lys25<br>Lys56-Glu52	Asp55(M)-Lys51(M)<br>Asn4(S)-Gln23(S)<br>Ser32(S)-Cys37(M)<br>Ser6(M)-Cys21(M)	-
III	2α + 2β	Zn_4MT2	-	Lys44(S)-Cys59(M)<br>Gln46(S)-Ala42(M)<br>Asn4(S)-Gln23(S)<br>Ser12(S)-Cys26(S)	Lys43-Cys59<br>Lys43-Cys60
V	3α + 2β	Zn_5MT2	-	Ser18(M)-Cys15(M)<br>Cys29(M)-Cys26(M)<br>Gln23(S)-Ser32(M)	Lys31-Cys19
VI	4α + 2β	Zn_6MT2	-	Gln23(M)-Asn4(M)<br>Cys41(M)-Pro38(M)<br>Cys24(M)-Lys31(M)<br>Cys5(M)-Asp2(S)<br>Ser6(M)-Cys21(S)	Lys31-Cys19
VII	4α + 3β	Zn_7MT2	-	Lys25(S)-Ser45(S)<br>Ser54(M)-Lys51(M)<br>Asn4(S)-Gln24(S)	Lys50-Cys58<br>Lys31-Cys19

REFERENCES

1 Krężel, A.; Maret, W. Dual Nanomolar and picomolar Zn(II) binding properties of metallothionein. *J. Am. Chem. Soc.* **2007**, *129*, 10911–10921

2 Drozd, A.; Wojewska, D.; Peris-Díaz, M. D.; Jakimowicz, P.; Krężel, A. Crosstalk of the structural and zinc buffering properties of mammalian metallothionein-2. *Metallomics* **2018**, *10*, 595–613.

3 Eyer, P.; Worek, F.; Kiderlen, D.; Sinko, G.; Stuglin, A.; Simeon-Rudolf, V.; Reiner, E. Molar absorption coefficients for the reduced Ellman reagent: reassessment. *Anal. Biochem.* **2003**, *312*, 224–227.

4 Peris-Díaz, M. D.; Guran, R.; Zitka, O.; Adam, V.; Krężel, A. Metal- and affinity-specific dual labeling of cysteine-rich proteins for identification of metal-binding sites. *Anal. Chem.* **2020**, *92*, 12950–12958.

5 Alderighi, L.; Gans, P.; Ienco, A.; Peters, D.; Sabatini, A.; Vacca, A. Hyperquad simulation and speciation (HySS): a utility program for the investigation of equilibria involving soluble and partially soluble species. *Coord. Chem. Rev.* **1999**, *184*, 311–318.

6 Marty, M. T.; Baldwin, A. J.; Marklund, E. G.; Hochberg, G. K. A.; Benesch, J. L. P.; Robinson, C. V.; Bayesian deconvolution of mass and ion mobility spectra: from binary interactions to polydisperse ensembles. *Anal. Chem.* **2015**, *87*, 4370–4376.
